# Sec16A is critical for both conventional and unconventional secretion of CFTR

**DOI:** 10.1038/srep39887

**Published:** 2017-01-09

**Authors:** He Piao, Jiyoon Kim, Shin Hye Noh, Hee-Seok Kweon, Joo Young Kim, Min Goo Lee

**Affiliations:** 1 Department of Pharmacology, Brain Korea 21 PLUS Project for Medical Sciences, Severance Biomedical Science Institute, Yonsei University College of Medicine, Seoul 03722, Korea; 2 Division of Electron Microscopic Research, Korea Basic Science Institute, Daejeon 34133, Korea

## Abstract

CFTR is a transmembrane protein that reaches the cell surface via the conventional Golgi mediated secretion pathway. Interestingly, ER-to-Golgi blockade or ER stress induces alternative GRASP-mediated, Golgi-bypassing unconventional trafficking of wild-type CFTR and the disease-causing ΔF508-CFTR, which has folding and trafficking defects. Here, we show that Sec16A, the key regulator of conventional ER-to-Golgi transport, plays a critical role in the ER exit of protein cargos during unconventional secretion. In an initial gene silencing screen, Sec16A knockdown abolished the unconventional secretion of wild-type and ΔF508-CFTR induced by ER-to-Golgi blockade, whereas the knockdown of other COPII-related components did not. Notably, during unconventional secretion, Sec16A was redistributed to cell periphery and associated with GRASP55 in mammalian cells. Molecular and morphological analyses revealed that IRE1α-mediated signaling is an upstream regulator of Sec16A during ER-to-Golgi blockade or ER stress associated unconventional secretion. These findings highlight a novel function of Sec16A as an essential mediator of ER stress-associated unconventional secretion.

The cystic fibrosis (CF) transmembrane conductance regulator (CFTR) is an N-glycosylated transmembrane protein with anion channel activity that permeates chloride and bicarbonate at the apical surface of secretory epithelia of the airways, intestine, pancreas, and exocrine glands^1^,^2^. Loss-of-function mutations in CFTR are associated with CF and several other human diseases of the epithelial organs, such as bronchiectasis and chronic pancreatitis^3^,^4^. CFTR is synthesized in the endoplasmic reticulum (ER) and transported to the cell surface via the conventional Golgi-mediated secretion pathway. Thus, the Golgi-matured, fully N-glycosylated CFTR is expressed at the cell surface^5^. The most common disease-causing mutation of CFTR, a phenylalanine deletion at position 508 (ΔF508), results in protein misfolding and retention in the ER, leading to defects in the cell-surface expression of CFTR^6^. As a result, negligible amounts of ΔF508-CFTR reach the plasma membrane, and ΔF508-CFTR remains in a core-glycosylated immature form within the ER^5^.

Interestingly, under ER-to-Golgi blockade or ER-stress conditions, core-glycosylated wild-type and ΔF508 CFTR in the ER can travel to the cell surface through an unconventional Golgi reassembly stacking protein (GRASP)-dependent pathway that bypasses the Golgi^7^. Furthermore, augmentation of this unconventional secretion pathway via GRASP55 overexpression has been shown to rescue the defects caused by ΔF508-CFTR in a murine CF model^7^. However, molecular mechanisms underlying the rescue, and especially the export of the ER-retained, core-glycosylated CFTR from the ER, remain elusive.

Under normal conditions, the export of secretory proteins from the ER is mediated by coat protein complex (COP) II-coated vesicles that bud from specific locations on the ER membrane called ER exit sites (ERES) or transitional ER^8^. COPII assembly begins with the Sec12-catalyzed activation of the small GTPase Sar1^9^, followed by the sequential recruitment of Sec23–24 dimer and Sec13–31 dimer lattice assembly to form the inner and outer layers of the COPII coat, respectively^10^,^11^. In addition to these core COPII molecules, Sec16 plays an essential role in the COPII-mediated exit of protein cargos from the ER in organisms ranging from yeast to mammals. Sec16 is a large, peripheral membrane protein that is tightly associated with ERES and is proposed to mediate ERES biogenesis and act as a scaffold for COPII assembly by interacting with multiple COPII components (Sec23, Sec24, Sec13, and Sec31), as well as with Sar1-GTPase^12^,^13^,^14^. Two orthologous genes encode the human Sec16 (Sec16A and Sec16B), and among them Sec16A appears to be the primary ortholog, because it is the most similar to the Sec16 proteins of other species (~240 KDa, in size).

Several cellular signals regulate COPII-mediated protein transport and generation of ERES via modulating Sec16. For example, ERK-2 regulates the number of ERES by modulating Sec16 phosphorylation^15^. In addition, inositol-requiring enzyme 1 (IRE1), a transducer of ER stress signals and the unfolded protein response (UPR)^16^, was shown to regulate Sec16A^17^. During protein overload in the ER lumen, the number of ERES increases together with the Sec16A levels in response to the increased cargo load^17^. Notably, the IRE1-mediated signaling is also required for the unconventional, ER stress-associated secretion of CFTR^7^. The blockade of ER-to-Golgi transport, either direct via the inhibition of COPII-mediated vesicular transport (e.g., transfection with the dominant-negative form of Sar1), or indirect via the inhibition of COPI-mediated transport (e.g., transfection with the dominant-negative form of Arf1), triggers the activation of ER stress^18^ and evokes the unconventional secretion of core-glycosylated CFTR via the GRASP-dependent mechanism in mammalian cells^7^.

In an initial RNA interference (RNAi) screen of COPII-associated components, we found that Sec16A knockdown abolished the unconventional secretion of wild-type and ΔF508 CFTR induced by ER-to-Golgi blockade, whereas the knockdown of other COPII-related components did not. We then examined the role of Sec16A in the unconventional secretion pathway and found that Sec16A is a critical component in the ER stress-associated, GRASP-mediated unconventional secretion of core-glycosylated CFTR. In addition, we found that IRE1-mediated signaling is an upstream regulator of Sec16A during ER stress-induced unconventional secretion. Our results provide new insights into the global role of Sec16A as a common mediator of both the conventional and the unconventional export of secretory cargos from the ER.

## Results

### Sec16A is required for both conventional and unconventional secretion of CFTR

**Figure 1:**
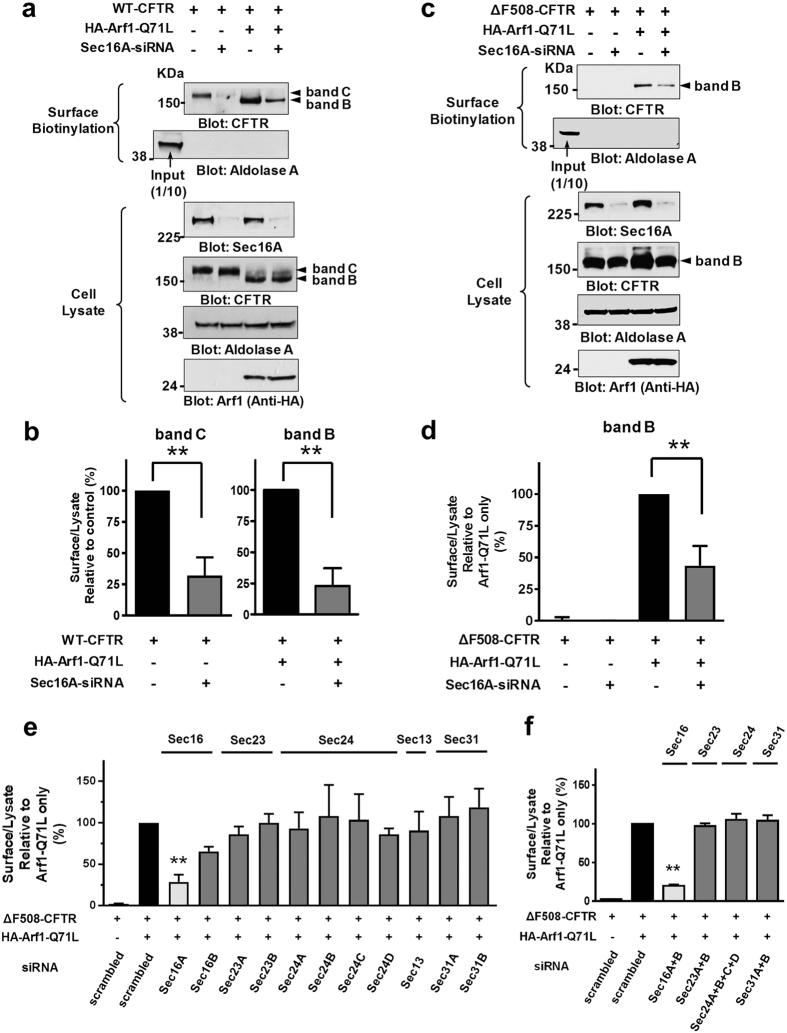
Sec16A, but not core COPII components, is required for unconventional secretion of CFTR. (**a–d**) Surface biotinylation of CFTR. HEK293 cells were transfected with plasmids expressing wild-type (WT) (**a**,**b**) or ΔF508 CFTR (**c**,**d**), and a surface biotinylation assay was performed 24 h after transfection. Some cells were cotransfected with the Arf1-Q71L plasmid to induce ER-to-Golgi blockade. The cells were pretreated with scrambled or Sec16A-specific siRNAs (100 nM) 24 h before plasmid transfection. Cell surface-specific labeling of proteins was confirmed by the absence of the cytosolic protein aldolase A in the biotinylated fraction. Representative surface biotinylation assays are shown (**a**,**c**), and the results of multiple experiments (n = 4) are summarized (**b**,**d**). Sec16A knockdown abolished both the conventional cell-surface trafficking of the Golgi complex-glycosylated WT-CFTR (band C) and the unconventional cell-surface trafficking of the ER core-glycosylated WT, as well as ΔF508 CFTRs (band B) induced by Arf1-Q71L. (**e,f**) Effects of COPII depletion on the Arf1-Q71L-induced cell-surface expression of ΔF508-CFTR. Effects of individual knockdown of each isoform are shown in (**e**) and those of combinatorial knockdown of the same gene family are shown in (**f**). The results of multiple experiments (n = 3–7) are summarized, and images of representative surface biotinylation assays are shown in Figure S2. Knockdown of Sec16B and core COPII components (Sec23, Sec24, Sec13, and Sec31) did not affect the unconventional cell-surface transport of ΔF508-CFTR. The depletion of Sec16A and COPII components by siRNA was confirmed by immunoblotting or mRNA quantitation (Figure S3). Data are shown as mean ± SEM. ns: not significant, **P < 0.01.

Wild-type CFTR undergoes Golgi-mediated complex glycosylation, and the complex-glycosylated CFTR (Fig. 1, band C) was expressed at the plasma membrane in a surface biotinylation assay (Fig. 1a). As reported previously^7^, the induction of ER-to-Golgi blockade by the dominant-negative Arf1-Q71L mutant induced the cell-surface expression of ER core-glycosylated wild-type CFTR and also the folding-deficient ΔF508-CFTR via unconventional protein secretion (Fig. 1a–d, lane 3). To exclude the possibility that these results were caused by a nonspecific inhibition of membrane protein internalization, we repeated the experiments with the inhibitor of dynamins (dynasore) or the dominant-negative dynamin mutant (dynamin2-K44A) that had been shown to inhibit the internalization of plasma membrane proteins, such as transferrin receptor and CFTR^19^,^20^. As shown in Figure S1, although these treatments increased the cell-surface expression of transferrin receptor and complex-glycosylated wild-type CFTR by 40–80% (Figure S1a,b), dynasore and dynamin2-K44A neither affected Arf1-Q71L-mediated cell-surface expression of core-glycosylated CFTR nor induced cell-surface expression of ΔF508-CFTR (Figure S1b–d).

We then explored the role of COPII-associated components in unconventional ER secretion using an RNAi screen in HEK293 cells (Fig. 1 and Figure S2). Notably, depletion of Sec16A abolished the cell-surface expression of CFTR mediated by both the conventional and the unconventional secretion pathways. Sec16A silencing by small interfering RNA (siRNA) reduced the surface expression of complex-glycosylated CFTR (Fig. 1a,b lane 2). Moreover, Sec16A depletion strongly diminished the unconventional cell-surface expression of wild-type CFTR (Fig. 1a,b, lane 4) and also interfered with the cell-surface expression of ΔF508-CFTR induced by Arf1-Q71L (Fig. 1c,d, lane 4). However, depletion of Sec16B and core COPII components, such as Sec23, Sec24, Sec13, and Sec31, did not affect the unconventional cell-surface transport of ΔF508-CFTR (Fig. 1e,f and Figure S2). Immunoblotting and mRNA quantitation confirmed the effects of RNAi on the depletion of Sec16 and core COPII components (Figure S3).

**Figure 2:**
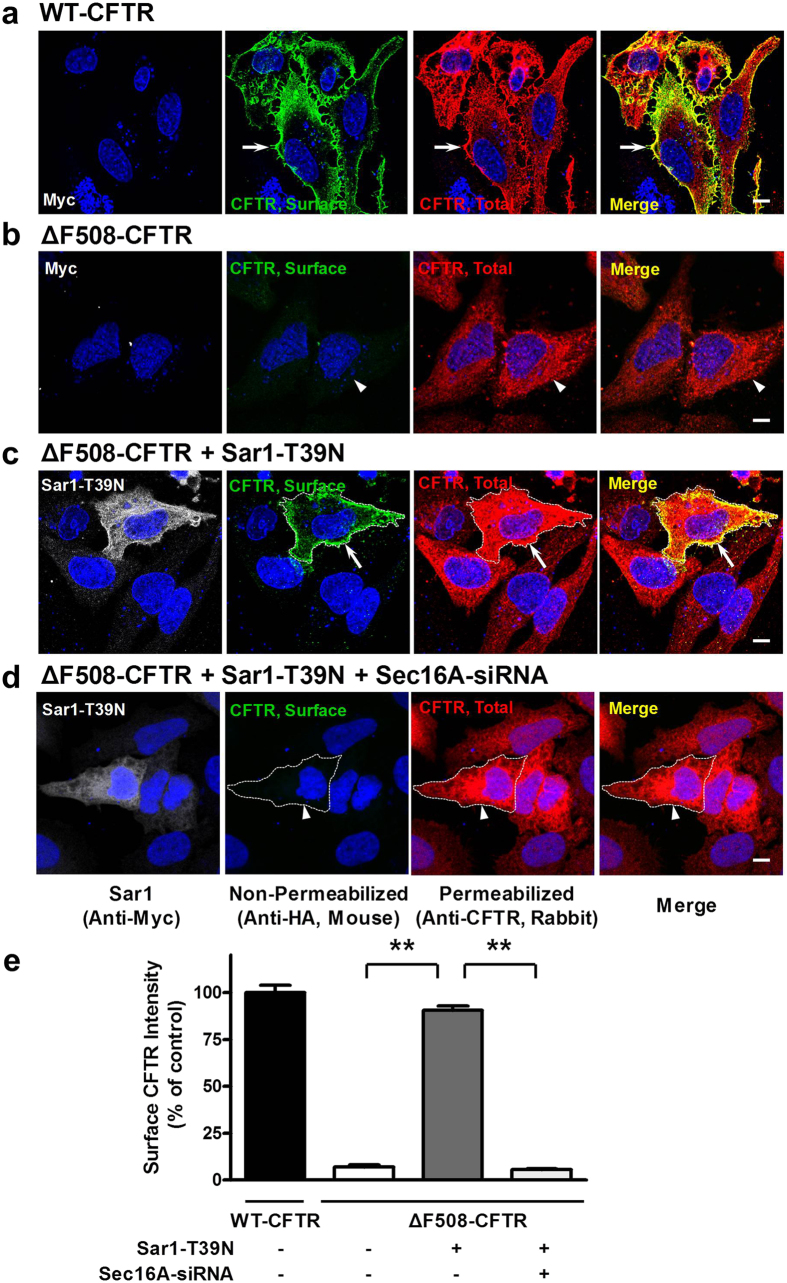
Sec16A silencing inhibits unconventional ΔF508-CFTR trafficking to the cell surface. (**a–d**) Representative immunofluorescence images of wild-type (WT) or ΔF508-CFTR. Extracellular loop HA-tagged WT (**a**) and ΔF508 (**b–d**) CFTRs were expressed in HeLa cells. CFTR at the cell surface was immunostained with anti-HA antibodies before membrane permeabilization (green), and then the total CFTR was stained with anti-R4 CFTR antibodies after permeabilization (red). Some cells coexpressed Myc-Sar1-T39N to induce unconventional surface expression of ΔF508-CFTR (**c,d**). The expression of the Sar1-T39N mutant was confirmed by anti-Myc staining. The cells were pretreated with scrambled (**b,c**) or Sec16A-specific (**d**) siRNAs (100 nM) 24 h before plasmid transfection. Cells expressing Sar1-T39N are marked with white dotted lines. Arrows indicate surface expression of WT or ΔF508 CFTRs and arrowheads indicate cells that do not express surface CFTR. (**e**) Quantification of surface CFTR intensity. Data are shown as mean ± SEM from three independent experiments (each comprising analyses of 20–30 cells). Sec16A depletion abolished the cell-surface expression of ΔF508-CFTR induced by Sar1-T39N. Scale bar: 10 μm, **P < 0. 01.

The effects of Sec16A depletion on the cell-surface expression of CFTR were further examined using immunostaining in mammalian cells. HeLa cells were used instead of HEK293 cells for morphological analyses, because they attach more firmly to coverslips, which prevents the loss of cells and the derangement of cell shapes during immunofluorescence procedures^21^. HeLa cells were transfected with extracellular HA-tagged wild-type or ΔF508 CFTR in order to identify cell-surface CFTR under non-permeabilized conditions. Some cells were cotransfected with the GDP-restricted Sar1-T39N mutant to block COPII-mediated ER-to-Golgi transport^22^. In control cells, the wild-type CFTR was abundantly expressed on the plasma membrane (Fig. 2a), while the folding-deficient ΔF508-CFTR failed to reach the cell surface (Fig. 2b). The blockade of ER-to-Golgi transport by Sar1-T39N evoked the cell-surface expression of ΔF508-CFTR (Fig. 2c). Notably, depletion of Sec16A abolished the unconventional cell-surface expression of ΔF508-CFTR induced by Sar1-T39N (Fig. 2d,e). Taken together, the results indicate that Sec16A, but not core COPII components, is required for the unconventional secretion of CFTR.

Given the finding that GRASP55 overexpression alone was sufficient to activate the surface transport of ΔF508-CFTR^7^, we asked whether Sec16A is involved in the GRASP overexpression-induced unconventional secretion pathway. We cotransfected HEK293 cells with three different plasmids expressing GRASP55 (Myc-GRASP55, Myc-tagging at the N-terminus of GRASP55; GRASP55-Myc, Myc-tagging at the C-terminus of GRASP55; and GRASP55 without any tag), which were shown to rescue the cell-surface expression of ΔF508-CFTR^21^. Sec16A depletion by siRNA significantly reduced the ΔF508-CFTR surface trafficking induced by the overexpression of all three forms of GRASP55 (Figure S4), indicating that Sec16A is required for GRASP-mediated unconventional secretion of ΔF508-CFTR.

### Sec16A colocalizes and associates with GRASP during ER stress-associated unconventional secretion

**Figure 3:**
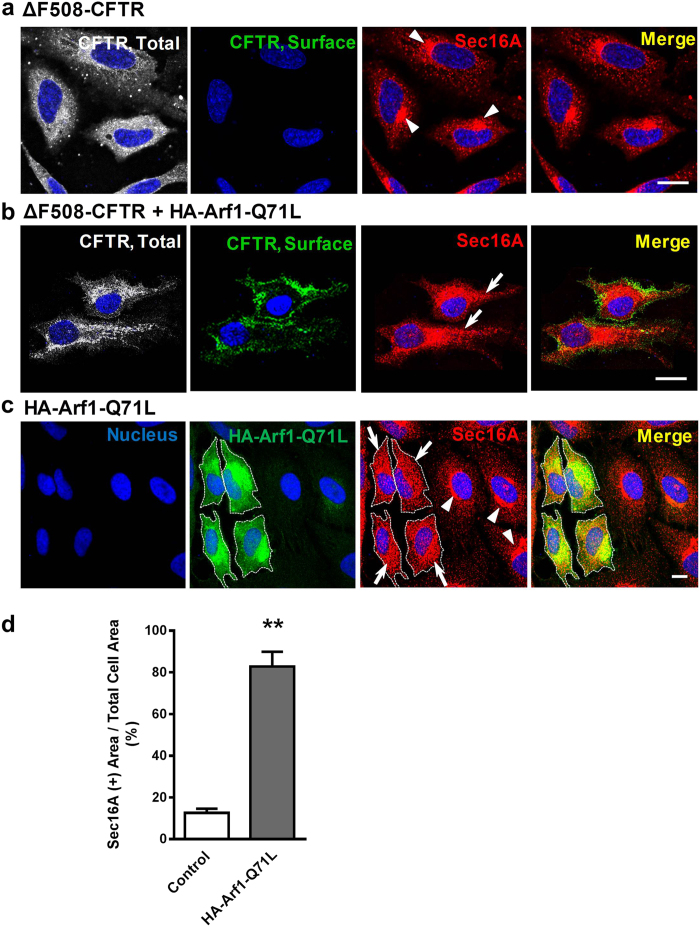
Relocalization of Sec16A in Arf1-Q71L-induced unconventional secretion of ΔF508-CFTR. (**a,b**) Intracellular localization of Sec16A was analyzed using immunocytochemistry. HeLa cells were transfected with plasmids expressing extracellular loop HA-tagged ΔF508-CFTR with or without Arf1-Q71L. CFTR at the cell surface was immunostained with anti-HA antibodies before membrane permeabilization (green). Then, the total CFTR was stained with anti-M3A7 CFTR antibodies (grey), and Sec16A was stained with anti-KIAA0310 Sec16A antibody (red). In control cells (**a**), Sec16A puncta were mostly localized in the juxtanuclear area (arrowhead). (**b**) ER-to-Golgi blockade by Arf1-Q71L evoked the cell-surface expression of ΔF508-CFTR (green) and the redistribution of Sec16A to entire cellular area (arrows, red). (**c,d**) Sec16A redistribution in cells coexpressing HA-Arf1-Q71L (white dotted line) was compared with that in cells not expressing HA-Arf1-Q71L. Representative immunofluorescence images are shown (**c**), and the results of multiple experiments (n = 5, each comprising analyses of 5–10 cells, **P < 0.01) are summarized (**d**). Scale bar: 5 μm, **P < 0.01.

We next investigated the relationship between Sec16A and cell-surface CFTR expression induced by ER-to-Golgi blockade to further identify the role of Sec16A in unconventional secretion. The blockade of ER-to-Golgi transport by Arf1-Q71L induced the cell-surface expression of ΔF508-CFTR in HeLa cells (Fig. 3a,b). Interestingly, Arf1-Q71L altered the intracellular localization of Sec16A. In control cells, Sec16A displayed typical ERES localization, in which Sec16A puncta were mostly concentrated in the juxtanuclear area (Fig. 3a and HA-negative cells in Fig. 3c; arrowheads). However, when the ER-to-Golgi transport was blocked by Arf1-Q71L, Sec16A dispersed into more peripheral regions and the Sec16A puncta appeared in >80% of the cytoplasmic area (Fig. 3b–d, arrows). These results are comparable to a recent finding that ER relocalization of GRASP is a requisite for unconventional secretion of CFTR^21^.

**Figure 4:**
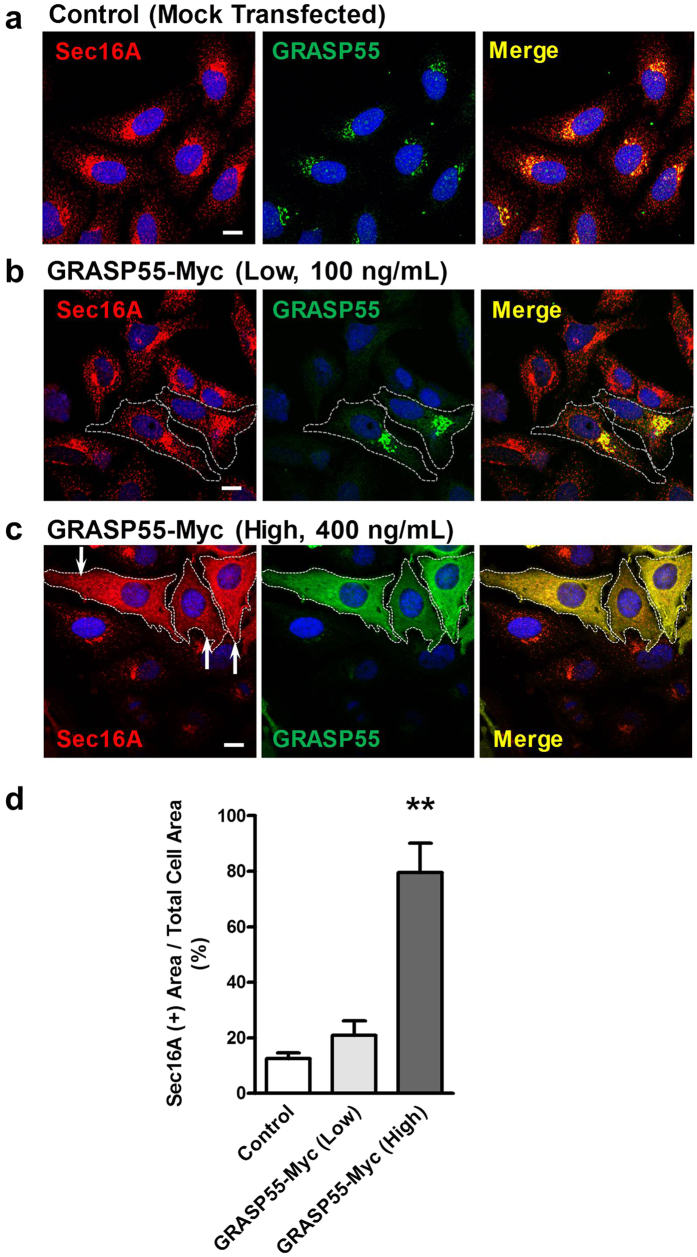
Relocalization of Sec16A in GRASP-mediated unconventional secretion of ΔF508-CFTR. (**a–c**) Cellular distribution of Sec16A was analyzed using immunocytochemistry. HeLa cells were transfected with mock or GRASP55-Myc plasmids. Representative immunofluorescence images are shown. Endogenous GRASP55 (**a**) and a low level of GRASP55-Myc (**b**), which does not induce unconventional secretion (Figure S5a), were expressed at the perinuclear Golgi apparatus, and Sec16A was enriched around that region. In contrast, Sec16A puncta and GRASP55 were redistributed to the entire cellular area in cells with a high level of GRASP55-Myc expression (**c**), which does induce unconventional secretion of ΔF508-CFTR (Figure S5b). (**d**) Quantification of the ratio of the Sec16A (+) area versus the total cell area in multiple experiments (mean ± SEM, n = 5, each comprising analyses of 5–10 cells) are summarized. Scale bar: 10 μm, **P < 0.01: difference from control.

**Figure 5:**
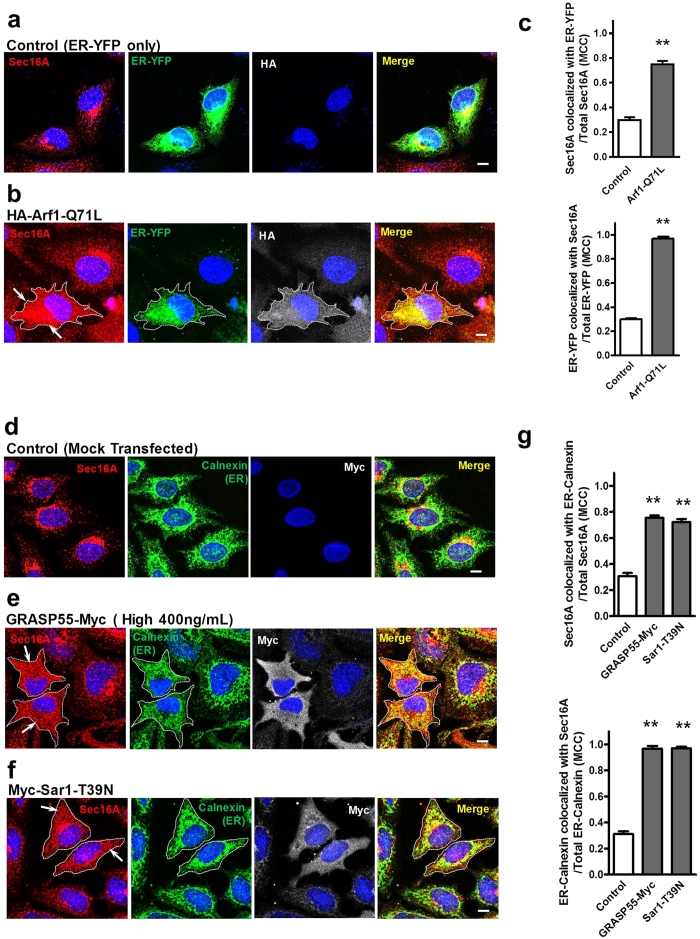
Relocalized Sec16A colocalizes with the ER marker proteins. (**a–c**) Cellular distribution of Sec16A was analyzed using immunocytochemistry in HeLa cells. Cells were transfected with plasmids encoding the ER marker protein ER-yellow fluorescent protein (ER-YFP) with or without Arf1-Q71L. Representative immunofluorescence images are shown (**a,b**), and the results of multiple experiments are summarized (**c**). Analyses using Manders’ colocalization coefficient (MCC) show that ER-to-Golgi blockade by Arf1-Q71L increased the extent of correlation between ER-YFP and Sec16A (**c**; mean ± SEM, n = 6, each comprising analyses of 5–10 cells). (**d–g**) Cells were transfected with mock (**d**), GRASP55-Myc (**e**), or Myc-Sar1-T39N plasmids (**f**), and the ER marker protein calnexin was co-immunostained with Sec16A. GRASP55-Myc and Myc-Sar1-T39N were immunostained with anti-Myc antibody. Representative immunofluorescence images are shown (**d–f**), and the results of multiple experiments are summarized (**g**). White dotted line shows cell periphery of GRASP55-Myc or Sar1-T39N expressing cells. Arrows indicated the redistribution of Sec16A to entire cellular area by ER-to-Golgi blockade or GRASP55 overexpression. The MCC analyses show that GRASP55 overexpression and ER-to-Golgi blockade by Sar1-T39N increased the extent of correlation between calnexin and Sec16A (**g**; mean ± SEM, n = 6, each comprising analyses of 5–10 cells). Scale bar: 10 μm. **P < 0.01: difference from each control.

Therefore, to investigate the relationship between Sec16A and GRASP55, we examined the cellular localization of GRASP55 and Sec16A. Endogenous GRASP55 and a low level of GRASP55-Myc, which does not induce unconventional secretion (Figure S5a), were expressed at the typical perinuclear Golgi apparatus, and Sec16A-positive ERES were enriched around that region to facilitate ER-to-Golgi transport (Fig. 4a,b). Notably, in HeLa cells with a high level of GRASP55-Myc expression, which does induce unconventional ΔF508-CFTR secretion (Figure S5b), the Sec16A puncta and GRASP55 were significantly redistributed to the entire cellular area (Fig. 4c, arrows). Subsequent immunofluorescence colocalization analyses assessing both the percentage of overlapping area and the pixel colocalization correlation between Sec16A and ER markers revealed that the relocalized Sec16A puncta were highly colocalized with the ER-marker proteins, suggesting that Sec16A relocalized to the peripheral ER area (Fig. 5). The peripheral localization of Sec16A increased Manders’ Colocalization Coefficient (MCC) due to an increment in the co-incident coefficient between the fractions of Sec16A and ER marker proteins (Fig. 5c,g).

**Figure 6:**
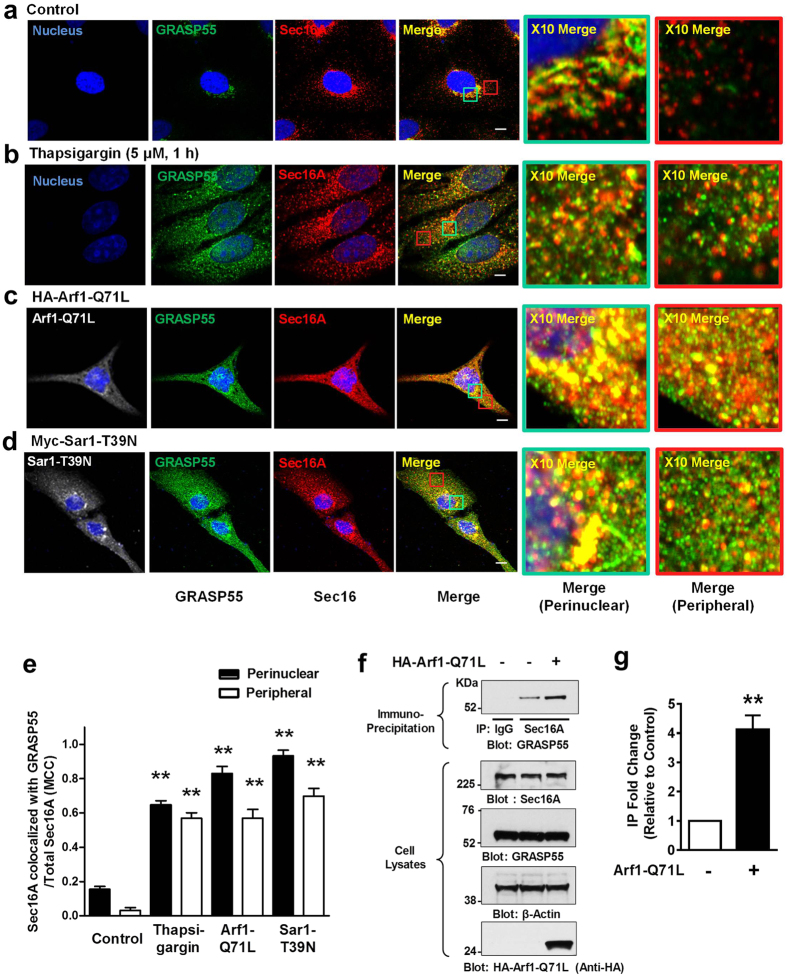
Sec16A colocalizes and interacts with GRASP55. (**a–d**) The cellular localization of Sec16A and GRASP55 was analyzed using immunocytochemistry in HeLa cells. Representative immunofluorescence images are shown. Endogenous GRASP55 was labeled with fluorophore-tagged antibodies (green), and Sec16A was labeled with fluorophore-tagged antibodies (red). Some cells were cotransfected with Arf1-Q71L (**c**) or Sar1-T39N (**d**) to induce ER-to-Golgi blockade. The induction of ER stress (thapsigargin) (**b**) or ER-to-Golgi blockade (Arf1-Q71L or Sar1-T39N) increased the colocalization of Sec16A and GRASP55 in both the perinuclear (green box) and the peripheral (red box) regions. (**e**) Analyses using Manders’ colocalization coefficient (MCC) show that ER stress (thapsigargin) and ER-to-Golgi blockade (Arf1-Q71L or Sar1-T39N) significantly increased the extent of correlation between Sec16A and GRASP55 in both the perinuclear and peripheral regions (mean ± SEM, n = 5, each comprising analyses of 5–10 cells). (**f,g**) Coimmunoprecipitation experiments with Sec16A and GRASP55 were performed in HEK293 cells. A representative coimmunoprecipitation assay is shown (**f**), and the results of multiple experiments (n = 3) are summarized (**g**). Protein samples were precipitated with anti-Sec16A (KIAA0310) and blotted with anti-GRASP55. ER-to-Golgi blockade by Arf1-Q71L increased the association between Sec16A and GRASP55. Scale bar: 10 μm, **P < 0.01: difference from each control.

Because Sec16A and GRASP55 showed similar cellular localization, we further examined their subcellular localization after the induction of unconventional secretion. The endogenous GRASP55 and Sec16A in HeLa cells were labeled with fluorophore-tagged antibodies. In control cells, although both GRASP55 and Sec16A were enriched in the juxtanuclear regions, analyses at higher magnifications revealed that the GRASP55 and Sec16A were mostly located in separate regions (Fig. 6a). In addition, Sec16A puncta located in the cell periphery were completely devoid of GRASP55 (Fig. 6a). Stimuli that induce unconventional secretion, such as ER stress and ER-to-Golgi blockade, significantly redistributed and colocalized the Sec16A and GRASP55. Treatment with thapsigargin, which induces ER stress by depleting Ca^2+^ stores in the ER lumen, and coexpression with Arf1-Q71L or Sar1-T39N, which induces ER-to-Golgi blockade, redistributed GRASP55 from the juxtanuclear region to entire cellular area, significantly increasing the overall colocalization of GRASP55 with Sec16A puncta, especially in the peripheral regions (Fig. 6b–e). Electron microscopy further supported the finding that Arf1-Q71L-induced ER-to-Golgi blockade redistributed the Golgi protein GRASP55 from the Golgi to the ER, where Sec16A is localized (Figure S6).

To elucidate the platform role of Sec16A in the GRASP-mediated unconventional trafficking pathway, we performed an immunoprecipitation assay to assess the protein-protein interaction between GRASP55 and Sec16A. In HEK293 cells transfected with mock plasmids, the interaction between endogenous GRASP55 and Sec16A was minimal under control conditions. Notably, the interaction became much stronger, when ER-to-Golgi blockade was induced by Arf1-Q71L (Fig. 6f,g). Taken together, the results suggest that ER stress and ER-to-Golgi blockade facilitate the assembly of large complexes containing GRASP55 and Sec16A.

### ER-to-Golgi blockade and GRASP55 overexpression do not relocalize Sec31A, a core COPII component

**Figure 7:**
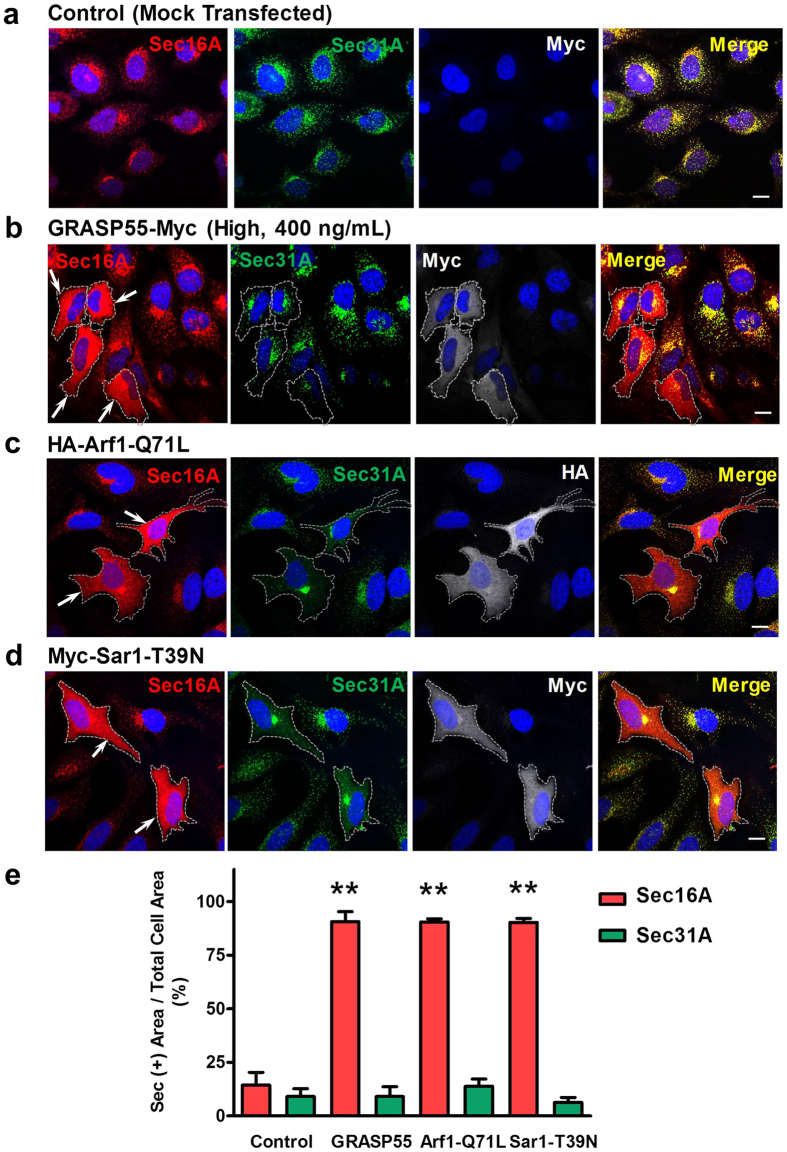
ER-to-Golgi blockade and GRASP55 overexpression do not relocalize Sec31A. (**a–d**) The cellular localization of Sec31A, a core COPII component, was compared with that of Sec16A using immunocytochemistry in HeLa cells. Representative immunofluorescence images are shown. Some cells were cotransfected with GRASP55-Myc (**b**), Arf1-Q71L (**c**), or Sar1-T39N (**d**) to induce Sec16A redistribution. (**e**) Quantification of the ratio of the Sec16A (+) or Sec31A (+) area versus the total cell area in multiple experiments (mean ± SEM, n ≥ 5, each comprising analyses of 5–10 cells) are summarized. GRASP55 overexpression, Arf1-Q71L, or Sar1-T39N-induced ER-to-Golgi blockade caused no significant alterations of Sec31A localization. Scale bar: 10 μm, **P < 0.01: difference from each control.

To determine whether Sec16A relocalization by GRASP overexpression or ER-to-Golgi blockade is a generalized phenomenon of the COPII machinery, we examined the subcellular localization of Sec31A, a representative subunit of COPII (Fig. 7). Again, a high level of GRASP55 expression or ER-to-Golgi blockade by Sar1-T39N relocalized the Sec16A puncta into >80% of the whole cellular area in HeLa cells, whereas only ~20% of the cellular area was Sec16A-positive in control cells. In contrast, neither GRASP55 expression nor ER-to-Golgi blockade by Arf1 and Sar1 mutants significantly altered the Sec31A distribution. Morphometric quantification indicated that Sec31A remained in ~15% of the whole cellular area of cells with GRASP55 overexpression or Sar1-T39N (Fig. 7). Interestingly, the cellular distribution of Sec31A seemed to be contracted by the ER-to-Golgi blockade, especially by Sar1-T39N, although the change was not statistically significant (Fig. 7c–e). Coimmunostaining with γ-tubulin revealed that Sec31A became highly concentrated at the minus ends of the microtubules, known as the centrosome or the microtubule-organizing center (MTOC; Figure S7). In addition, the distribution of Sec24, another core component of COPII, was also not affected by ER-to-Golgi blockade (Figure S8). Collectively, the results suggest that the formation of GRASP-mediated unconventional vesicles is accompanied by the assistance of the ERES organizer/regulator protein Sec16A, but is independent of the core COPII components.

### IRE1α regulates unconventional secretion of ΔF508-CFTR via Sec16A modulation

**Figure 8:**
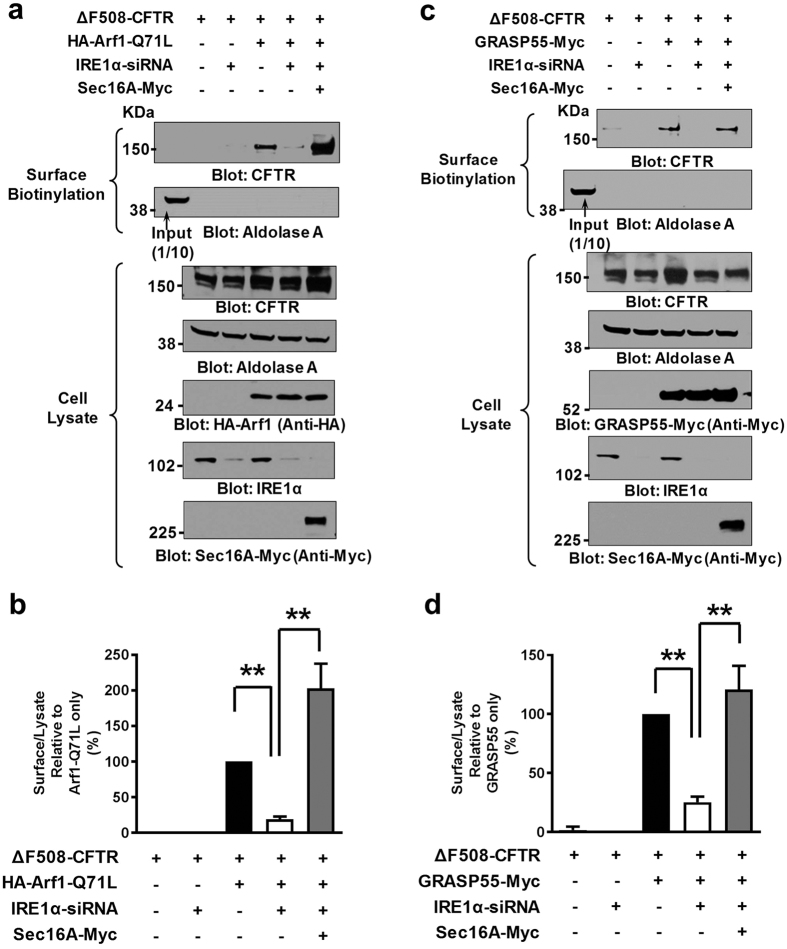
IRE1α depletion inhibits unconventional ΔF508-CFTR secretion by down-regulating Sec16A. (**a,c**) Surface biotinylation of CFTR was performed in HEK293 cells after the induction of unconventional ΔF508-CFTR secretion by Arf1-Q71L (**a**) or by GRASP55 overexpression. (**c**) The cells were pretreated with scrambled or IRE1α-specific siRNAs (100 nM) 24 h before plasmid transfection. (**b,d**) The results of multiple experiments (n = 3) are summarized. IRE1α depletion by siRNA inhibited the Arf1-Q71L-induced or GRASP55-induced surface expression of ΔF508-CFTR. Supplementation with exogenous Sec16A rescued the effects of Arf1-Q71L and GRASP55, which induced the cell-surface expression of ΔF508-CFTR. **P < 0.01.

**Figure 9:**
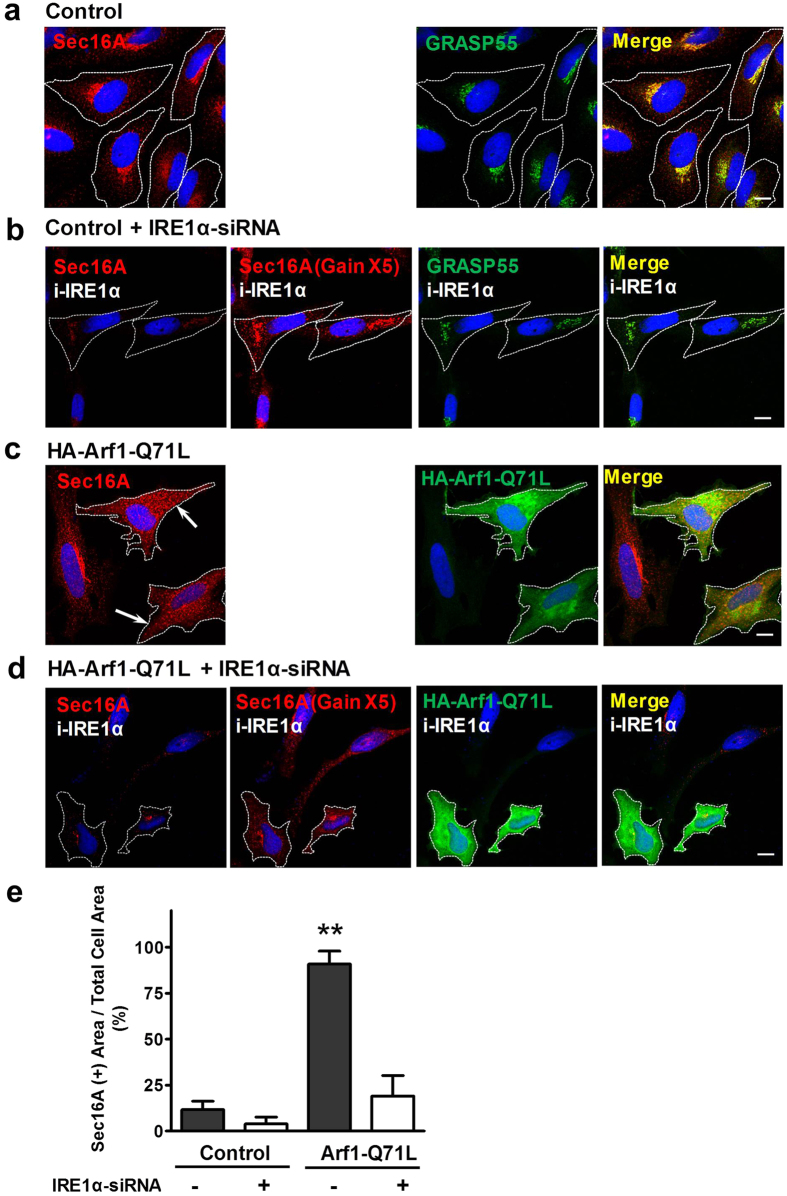
IRE1α depletion inhibits Sec16A redistribution. (**a–d**) The cellular localization of Sec16A was analyzed using immunocytochemistry in control HeLa cells (**a,b**) and in cells after the induction of unconventional ΔF508-CFTR secretion by Arf1-Q71L (**c,d**). The cells were pretreated with scrambled or IRE1α-specific siRNAs (100 nM) 24 h before plasmid transfection. The intracellular distribution of Sec16A in IRE1α-depleted cells was analyzed using confocal images taken with five times higher acquisition gain (Gain X5) because IRE1α depletion reduced the overall Sec16A expression levels (**b,d**). (**e**) The results of multiple experiments (n ≥ 5, each comprising analyses of 5–10 cells) are summarized. IRE1α depletion inhibited the Arf1-Q71L-induced redistribution of Sec16A. Scale bar: 10 μm, **P < 0.01: difference from control.

IRE1-mediated signaling, which is required for ER stress-mediated and GRASP-mediated unconventional secretion of ΔF508-CFTR^7^, has been shown to induce the neo-generation of Sec16A and increase both the number and the size of ERES to relieve the protein burden in the ER during a chronic overload of secretory cargo^17^. Therefore, we next investigated the role of IRE1 in Sec16A-mediated unconventional secretion. As shown in Fig. 8a,b, the depletion of IRE1α by siRNA strongly inhibited Arf1-Q71L-induced surface expression of ΔF508-CFTR in HEK293 cells, but supplementation with exogenous Sec16A rescued the effect of mutant Arf1 that induced the cell-surface expression of ΔF508-CFTR. Furthermore, consistent with the mutant Arf1 results, GRASP55-induced ΔF508-CFTR rescue was inhibited by IRE1 silencing and recovered by Sec16A replenishment (Fig. 8c,d). Immunocytochemistry results further supported the notion that IRE1 is an upstream regulator of Sec16A expression and localization during unconventional secretion. IRE1α depletion reduced the overall Sec16A expression levels in HeLa cells, and the increased Sec16A-positive area resulting from Arf1-Q71L or GRASP55 overexpression was diminished from 91% to 19% (Fig. 9).

## Discussion

Most membrane and secretory proteins exit the ER through cargo sorting and COPII-coated vesicle formation at specific locations on the ER membrane called ERES, and target to the Golgi complex and subsequently to the plasma membrane^8^,^23^. In addition to this well-defined conventional trafficking pathway, recent evidence suggests that many cytosolic, nuclear, secretory, and membrane proteins reach the cell surface by unconventional secretion pathways that bypass the Golgi^24^. In general, unconventional secretion events are not constitutive, but are instead stress induced. Cellular stress signals such as inflammation, starvation, mechanical stress during development, and ER stress can induce the unconventional secretion of cytoplasmic and membrane proteins^7^,^25^,^26^. However, the mechanisms and molecular components of the unconventional pathways, and particularly, how the secretory cargos leave the ER are still not fully understood. Here, we provide evidence that Sec16A is involved in the ER exit of the cargos during ER stress-associated unconventional secretion in mammalian cells.

The requirement of Sec16A for the unconventional surface expression of CFTR is rather surprising, because Sec16A is regarded as one of the critical molecules in conventional ER-to-Golgi transport, and the inhibition of ER-to-Golgi transport in general activates unconventional secretion. Sec16A in mammals is a large hydrophilic protein that is tightly associated with ERES and makes an indispensable contribution to the organization of ERES^27^ and COPII-mediated ER export^28^,^29^. The inhibition of COPII function by Sar1-T39N induces, rather than inhibits, unconventional CFTR secretion (Fig. 2c). In contrast to that of Sar1 (Fig. 2c) and core COPII components (Fig. 1e and Figure S2), the silencing of Sec16A inhibited the unconventional secretion of CFTR induced by ER-to-Golgi blockade or GRASP55 overexpression (Figs 1, 2 and Figures S2 and S4). These results indicate that Sec16A is a global organizer or regulator of ER export with a role that is not restricted to the conventional COPII-mediated transport pathway.

Sec16 was initially identified as a potentiating factor in COPII vesicle budding in yeasts^14^. Subsequently, Sec16 analogs were found in other species and shown to be involved in the COPII-mediated ER exit of cargo proteins in those organisms. However, the specific and precise role of Sec16 in COPII-mediated transport is still not fully established. For example, Stephens *et al*. suggested that Sec16 is a central organizer of COPII vesicles that defines ERES localization and recruits core COPII components in a stoichiometric fashion^27^,^30^. In contrast, Glick *et al*. proposed that Sec16A regulates COPII turnover and consequently influences the stability of ERES instead of organizing COPII vesicle formation^31^. Although there is some debate about whether Sec16 recruits COPII to ERES or the other way around^31^, it is generally agreed that Sec16 associates with core COPII components and is required for export-site assembly and function^32^. Our results indicate that Sec16 is also required for the COPII-independent ER exit of secretory cargos. Considering the scaffolding role of Sec16, it will be intriguing to identify the binding partners of Sec16A during ER stress or ER-to-Golgi blockade. Such investigations should aid our understanding of the molecular components and the big picture of ERES for unconventional protein secretion.

A notable finding of the present study is the relocalization of Sec16A. In Drosophila larval muscle cells, it has been reported that parts of dGRASP colocalize with Sec16 and participate in the ER exit of the integrin subunit αPS2^33^. However, it is unclear as to whether dGRASP and Sec16 mediate unconventional secretion of αPS2 in Drosophila. Furthermore, dGRASP and Sec16 are enriched around the nucleus in Drosophila muscle cells during αPS2 secretion^33^. In contrast, GRASP55 and Sec16A relocalize to the cell periphery and mediate unconventional secretion of CFTR upon ER stress or ER-to-Golgi blockade in mammalian cells (Figs 3, 4, 5, 6 and 9). Notably, the core COPII component Sec31 did not relocalize under those conditions (Fig. 7). Instead of dispersing Sec31, Sar1-T39N seemed to cause Sec31 to move to a more central distribution relative to the MTOC (Figure S7), suggesting the possibility that there is a signal that induces the relocalization of Sec31 into the minus end of microtubules during strong ER-to-Golgi blockade. The ER-Golgi interface and ERES are concentrated in the juxtanuclear region adjacent to the MTOC. The COPII vesicles that depart ERES initially travel towards the minus ends of the microtubules, which transport cargo proteins to the ER-Golgi intermediate compartment, and eventually to the Golgi^32^. After being processed in the Golgi, the secretory cargos leave the trans-Golgi network and travel towards the plus ends of the microtubules via vesicular trafficking to reach the cell surface. The peripheral movement of Sec16 puncta raises the possibility that ER stress signals separate Sec16A from the core COPII components by transporting Sec16A towards the plus ends of the microtubules. The unconventional secretion of CFTR induced by ER stress and ER-to-Golgi blockade is sensitive to N-ethylmaleimide (NEM) and nocodazole^7^, indicating that it requires vesicular trafficking via microtubules. The peripheral localization of Sec16A and ERES for unconventional secretion might facilitate the direct trafficking of secretory vesicles to the cell surface via short-distance, plus-end trafficking on microtubules that bypasses the Golgi.

In the present study, inhibition of ER-to-Golgi transport (Figs 1, 2, 3) or upregulation of GRASP55 (Figure S4) induced cell-surface expression of core-glycosylated CFTR. An alternative explanation of this finding would be that Arf1-Q71L or other potentially cytotoxic treatments may nonspecifically increase cell-surface expression of membrane proteins by inhibiting internalization of surface proteins rather than activation of a secretion event. As shown in Figure S1, the inhibition of membrane-protein internalization by dynasore and dynamin2-K44A did not evoke the cell-surface expression of core-glycosylated CFTR, suggesting that inhibition of protein internalization cannot account for the cell-surface expression of core-glycosylated CFTR during ER stress and ER-to-Golgi blockade. An interesting finding to note is that the inhibition of cell surface-protein internalization did not significantly affect CFTR transported unconventionally to the cell surface by Arf1-Q71L (Figure S1b–d), while it did increase the cell-surface expression of conventionally transported membrane proteins by 40–80% (Figure S1a,b). These results suggest that Arf1-Q71L inhibited the internalization of surface proteins. Considering that the folding-deficient membrane proteins that are transported to the cell surface remain under the control of peripheral protein quality control^34^, the inhibition of internalization would be an additional benefit that increases their retention time at the plasma membrane, although the inhibition of internalization alone is not capable of inducing the cell-surface expression.

In vertebrate models, GRASPs were shown to be involved in the unconventional surface transport of CFTR and the thrombopoietin receptor Mpl (myeloproliferative leukemia virus oncogene), in which ER core-glycosylated forms of membrane proteins are directly transported to the cell surface^7^,^35^,^36^. Sec16A was also required for the GRASP55 overexpression-induced cell-surface expression of ΔF508-CFTR (Figure S4), and Sec16A colocalized and associated with GRASP55 during ER stress-induced unconventional secretion (Fig. 6). As a scaffold for organizing ERES^37^, the Sec16A that is dispersed peripherally by ER stress signals may recruit GRASP55, which can then bring CFTR to newly established ERES for unconventional secretion. The reverse also seems possible, however, because the overexpression of GRASP55, which induces unconventional trafficking of ΔF508-CFTR but not ER stress signals^7^, evoked Sec16A colocalization and peripheral dispersion (Fig. 4e). In any case, the association between Sec16A, an organizer of ERES, and GRASP, a cargo-recruiting factor, will be a critical event for the initiation of unconventional secretion of CFTR.

Another important finding of our study is that IRE1α regulates unconventional secretion by modulating Sec16A. To maintain homeostasis in the ER lumen, the ER responds to an excess of unfolded proteins and relieves ER stress by triggering UPR signaling pathways^38^. Generally, UPRs reduce the protein load in the ER by decreasing the transcription and translation of secretory proteins, and by directing misfolded proteins into the ER-associated degradation pathway^39^. In addition, UPRs may activate the secretion of misfolded proteins via the unconventional route to relieve ER burden^7^. IRE1, one of the three major branches of UPR signaling^16^, was shown to be responsible for the generation of new ERES and the upregulation of Sec16A during chronic overload of a secretory cargo^17^. IRE1 was also shown previously to mediate ER stress-induced unconventional secretion of CFTR^7^. Our current findings suggest that the IRE1-mediated UPR is a major upstream signal that regulates the generation and anchoring of Sec16A at the ERES involved in ER stress-induced and GRASP overexpression-mediated unconventional secretion (Fig. 8).

IRE1α depletion reduced the overall expression of Sec16A and seemingly inhibited the peripheral dispersion of Sec16A puncta (Fig. 9). It is possible, however, that a simple reduction in Sec16A levels by IRE1 knockdown, rather than by true inhibition of Sec16A redistribution, would lower the Sec16A in immunofluorescence to subthreshold levels more profoundly in the peripheral regions than in the perinuclear region. Although we analyzed the immunofluorescence images with an increased laser gain to minimize the problem (Fig. 9), our results cannot completely rule out this possibility. In any case, profound reductions in peripheral Sec16A levels are associated with decreases in unconventional secretion of CFTR. Considering that peripheral redistribution of GRASP55 is critical for the unconventional secretion of CFTR^21^, and that GRASP55 associates with Sec16A during ER-to-Golgi blockade (Fig. 6), peripherally localized Sec16A would play an important role in the ER exit of cargos during ER-to-Golgi blockade. Therefore, accumulated evidence suggests that 1) GRASP is redistributed from the Golgi to the ER during ER stress and recognizes some cargos subjected for unconventional secretion, particularly membrane proteins such as CFTR that have a PDZ-binding motif at their C-terminus, and 2) GRASP associates with the Sec16A-containing protein complex, which delivers the cargos to the vesicles that depart from the ER membrane during unconventional secretion.

ER stress signals induce the phosphorylation of the GRASP55 C-terminus, which is essential for ER stress-induced unconventional secretion of ΔF508-CFTR^7^, and several kinases are known to regulate Sec16A function^15^,^40^. Recently, Joo *et al*. reported that ATG1/ULK-mediated phosphorylation at the Ser846 residue of Sec16A is essential for the COPII-mediated ER-to-Golgi trafficking under basal physiologic conditions^41^. Considering the finding that ATG1 is also involved in the ER stress-induced, unconventional trafficking of CFTR^7^, it will be an interesting task to investigate whether phosphorylation(s) at specific Sec16A loci is required for unconventional secretion. Increasing evidence indicates that autophagy components are involved in various kinds of unconventional trafficking. For instance, coronaviruses use nonlipidated, ATG8/LC3-positive vesicles for replication, which emerge from the ER by a COPII-independent mechanism^42^. However, this pathway is not dependent on ATG7^42^, whereas the ER stress-induced, unconventional trafficking of CFTR is dependent on both ATG7 and ATG8^7^. Therefore, it appears that diverse cargos, from viral particles to mammalian membrane proteins, employ several combinations of autophagy components for their travel to the cell surface after ER exit.

In conclusion, we demonstrated the role of Sec16A in ER exit during unconventional secretion. In addition, we showed that IRE1α acts as an upstream signal for ER stress-associated unconventional secretion by modulating Sec16A. These results provide new insights into the global role of Sec16A as a common mediator of ER export. Furthermore, the identification of Sec16A as a platform for the exit of folding-deficient proteins from the ER will contribute to therapeutic strategies for diseases resulting from defects in the transport of misfolded proteins.

## Materials and Methods

### Cell culture, plasmids, transfection, gene-silencing, antibodies, and chemicals

HEK293 and HeLa cells were maintained in Dulbecco’s modified Eagle’s medium (DMEM high glucose) containing 10% fetal bovine serum (FBS) and penicillin (50 IU/mL)/streptomycin (50 μg/mL; all from Invitrogen) in a 5% CO_2_ incubator at 37 °C. The mammalian expression plasmid Sec16A-Myc/DDK was purchased from OriGene (plasmid # RC223625). The plasmids encoding human ΔF508-CFTR, extracellular tagged HA-CFTR, HA-ΔF508-CFTR, Myc-GRASP55, Myc-Transferrin receptor, HA-Arf1-Q71L, and Myc-Sar1-T39N were described previously^7^. The plasmids encoding HA-GRASP55 and untagged GRASP55 were generated by cDNA subcloning into pCMV-SPORT6. The plasmid encoding Flag-tagged dynamin2-K44A was generated by a PCR-based site directed mutagenesis after constructing pcDNA3.1(+)-hDynamin2 using a cDNA fragment amplified from HEK293 cells (gene ID:1785, 2613 bp). pEYFP-ER plasmid was purchased from BD Biosciences (San Jose, CA, USA). Plasmid DNA transfection into HEK293 or HeLa cells was performed using the TransIT-X2 Dynamic Delivery System (Mirus Bio LLC). ON-TARGETplus human Sec16B-siRNA, Sec23A-siRNA, Sec23B-siRNA, Sec24A-siRNA, Sec24B-siRNA, Sec24C-siRNA, Sec24D-siRNA, Sec31B-siRNA and IRE1α-siRNA were purchased commercially (SMARTpool; Dharmacon, Lafayette, CO, USA). Double-stranded siRNAs against human Sec16A (targeting sequence: CCAGGTGTTTAAGTTCATCTA), against human Sec13 (targeting sequence: GCACTCATGTTACGAGGAA), and against human Sec31A (targeting sequence: AACAGACAAGTTCAGCATATT) were custom synthesized by Bioneer (Daejeon, Korea). Transfection of siRNA duplexes into the HEK293 cells was performed using Lipofectamine 2000 reagent (Invitrogen) according to the manufacturer’s protocol. Plasmid DNA delivery was performed 24 h after siRNA transfection.

The following antibodies were acquired commercially: anti-Sec16A (KIAA0310, Bethyl Laboratories, INC); anti-Sec31A (BD Transduction Laboratories); anti-Sec24B and anti-Sec24D (Bethyl Laboratories, INC); anti-IRE1α, anti-Myc and anti-HA, (Cell Signaling Technology, Danvers, MA); anti-γ-tubulin (Sigma); anti-Flag (Sigma); anti-GRASP55 rabbit polyclonal and anti-Calnexin mouse monoclonal (Abcam, Cambridge, MA); anti-GRASP55 monoclonal (Novus Biologicals, LLC); anti-Aldolase A and anti-ß-actin (Santa Cruz Biotechnology, SantaCruz, CA); and anti-CFTR M3A7 (Millipore, Billerica, MA). The anti-R4 polyclonal antibody raised against peptides corresponding to amino acids 1458–1471 of human CFTR was previously reported^43^. Thapsigargin and dynasore were commercially purchased (Sigma Aldrich).

### Immunoprecipitation, immunoblotting, and surface biotinylation

Immunoblotting and immunoprecipitation were performed as described previously^7^. Briefly, HEK293 cells, transfected with the appropriate plasmids, were homogenized in lysis buffer containing 25 mM Tris (pH 7.4), 1% (v/v) NP40, 150 mM NaCl, 5% glycerol, and 1 mM EDTA supplemented with protease inhibitor mixture (Roche, Applied Science, Mannheim, Germany). For co-immunoprecipitation, cell lysates (1,000 μg protein) were diluted to a final volume of 500 μL in lysis buffer and pre-cleared with control Agarose Resin (crosslinked 4% beaded agarose) for 2 h at 4 °C. To avoid co-elution of the antibody heavy and light chains that may have co-migrated with the GRASP55 bands, we immobilized 10 μg affinity-purified rabbit anti-Sec16A (KIAA0310) antibody or normal rabbit IgG (Santa Cruz Biotechnology, SantaCruz, CA) to AminoLink Plus Coupling Resin (Pierce Co-Immunoprecipitation Kit, Pierce, Rockford, IL, USA) at room temperature for 2 h. To precipitate endogenous Sec16A, pre-cleared protein complexes were collected with antibody-coupled resin overnight at 4 °C and then washed five times with the lysis buffer; the immune complexes were eluted with 30 μL elution buffer (pH 2.8) containing primary amine and 5× lane-marker sample buffer (0.3 M Tris•HCl, 5% SDS, 50% glycerol, lane-marker tracking dye; pH 6.8) containing 100 mM DTT. Protein bands were detected by enhanced chemiluminescence, and the densities of the bands were quantified using imaging software (Multi Gauge ver. 3.0; Fujifilm, Tokyo, Japan).

The surface biotinylation assay was performed as previously described^7^. Briefly, HEK293 cells were washed with PBS containing 0.1 mM CaCl_2_ and MgCl_2_ (PBS-CM plus) 24 h after the transfection with the appropriate plasmids, and then incubated in 1 mL biotin solution (0.3 mg/mL Sulfo-NHS-SS-Biotin in cold PBS-CM plus, Thermo Pierce) for 30 min at 4 °C. The cells were then incubated with quenching buffer containing 0.5% bovine serum albumin (BSA) for 10 min and washed three times with PBS. After cell lysis, the lysates were centrifuged at 16,000 *g* for 20 min at 4 °C. The resulting supernatants containing equal amounts of total protein were incubated with 200 μL 10% streptavidin agarose (Thermo Fisher Scientific). The streptavidin-bound, biotinylated proteins were centrifuged, washed five times with lysis buffer, and eluted in 2× SDS sample buffer. The subsequent steps of the biotinylation assay were the same as those of the immunoblotting procedure.

### Immunocytochemistry, confocal microscopic image acquisition, and morphometric analysis

Immunocytochemistry was performed as previously reported^7^. HeLa cells were cultured on 18 mm round coverslips and fixed and permeabilized with cold methanol (−20 °C) for 6 min. The fixed cells were washed three times with PBS and then incubated with blocking solution containing 5% serum from the species from which the secondary antibody was raised in (horse/donkey/goat serum), 1% BSA, and 0.1% gelatin in PBS for 1 h at room temperature. After the blocking step, the cells were stained by incubation with the appropriate primary antibodies, followed by fluorophore-conjugated secondary antibodies. In the case of surface-specific immunostaining, cells were fixed with 4% formaldehyde for 6 min at room temperature without permeabilization and then stained with primary and secondary antibodies. Fluorescence images were captured using a laser scanning confocal microscope (LSM 780; Carl Zeiss, Berlin, Germany) with a 63 × 1.4 numerical aperture oil objective lens.

Morphometric analysis of the captured confocal images was performed using the MetaMorph microscopy analysis software (version 7.1; Molecular Devices, Sunnyvale, USA). For the quantification of the images under each condition, 24-bit confocal images containing red, green, and blue components were converted into three 8-bit mono-channel images. For the quantification of surface CFTR intensity, pixels above the threshold level of 60 were defined as CFTR. The intensity profile of each single cell was presented as the standard deviation around the mean of the average intensity value for the entire region. For the analyses of the Sec16A distribution, the high-resolution images of Sec16A punctates were smoothed using a low-pass filter in MetaMorph, and pixels above the threshold level of 30 were defined as Sec16A (+). The regions of the total cell area were determined by the trace region tools of MetaMorph using the differential interference-contrast images of the cells, then the ratio of the Sec16A (+) area versus the total cell area was calculated.

The degree of colocalization between Sec16A and ER markers (or GRASP55) was quantified based on MCC (Manders’ Colocalization Coefficients)^44^ using the colocalization module of the ZEN 2012 software (black edition; Carl Zeiss, Berlin, Germany). For the measurement of MCC under each condition, pixels above the fluorescence threshold level of both channels (red and green) 50 were defined as the overlapping signals. Then, the average of MCC obtained from the regions of interest was calculated. For two target proteins, represented as Sec16A and ER, two different MCC values were calculated as:


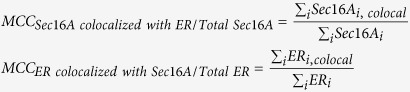


### Immunogold labeling and transmission electron microscopy

HeLa cells were fixed and subjected to cryoimmuno-EM labeling, as reported previously^45^. HeLa cells transfected with HA-Arf1-Q71L or empty plasmids were fixed with 4% paraformaldehyde and 0.1% glutaraldehyde in PBS buffer (pH 7.4) at 4 °C for 20 min. The fixed cells were embedded in 10% gelatin and cut into small blocks under a surgical microscope. The gelatin blocks were infused overnight with 2.3 M sucrose and frozen in liquid nitrogen. The frozen blocks were cut into 50 nm ultrathin cryosections at −120 °C using a Leica UCT7 cryo-ultramicrotome (Leica, Vienna, Austria). Ultrathin sections were transferred from the diamond knife onto Formvar-coated copper slot grids using a 2.3 M sucrose: 2% methylcellulose (1:1) solution. Rabbit polyclonal GRASP55 antibody (Abcam, diluted 1:50) was used as the primary antibody, and Protein A-10 nm gold conjugate (from Dept. of Cell Biology, University Medical Center Utrecht, Utrecht, Netherlands) was used to detect the primary antibody. Both the GRASP55 antibody and the Protein A-gold were diluted in 0.1% BSA-c (Aurion, Netherlands). For the immunogold labeling experiments, the ultrathin cryosections were first blocked with 0.1% cold fish gelatin and 5% BSA for 10 min, then incubated with GRASP55 antibody for 30 min, and continually with Protein A-gold for 30 min. The grids were stained in 4% neutral uranyl acetate and embedded in 2% methylcellulose containing 0.3% uranyl acetate, as previously described^46^. Transmission electron microscopy was conducted by examining the grids at 120 kV using a Tecnai G^2^ Spirit Twin Transmission Electron Microscope (FEI Company, USA).

### Quantitative real-time PCR (qPCR)

Purified RNA samples from HEK293 cells were reverse-transcribed using RNA to cDNA EcoDry Premixes (Takara Bio Inc.). The total reaction volume was adjusted to 20 μL with RNase-free water after mixing with 100 ng cDNA, 2 μL primer sets, 10 μL 2× SYBR premix Ex Taq, and 0.4 μL 50× ROX reference dye (Takara Bio Inc.). Amplification was performed under the following cycling conditions: 95 °C for 15 min, followed by 40 cycles of 95 °C for 15 s, and 60 °C for 40 s. Analyses were performed in triplicate for each cDNA. The relative mRNA expression levels were calculated using the comparative threshold cycle (C_t_) method with GAPDH as a control, as follows: ΔC_t_ = C_t_ (GAPDH) − C_t_ (target gene). The fold-change in gene expression normalized to GAPDH and relative to the control sample was calculated as 2^−ΔΔCt^.

### Statistical analysis

The results of multiple experiments are presented as the means ± SEM. Statistical analysis was performed using Student’s t-tests or with analysis of variance followed by Dunnet’s multiple comparison tests as appropriate; *P* < 0.05 was considered statistically significant. Calculations were performed using GraphPad Prism5 (GraphPad Software, Inc., La Jolla, CA).

## Additional Information

**How to cite this article**: Piao, H. *et al*. Sec16A is critical for both conventional and unconventional secretion of CFTR. *Sci. Rep.* **7**, 39887; doi: 10.1038/srep39887 (2017).

**Publisher's note:** Springer Nature remains neutral with regard to jurisdictional claims in published maps and institutional affiliations.

## Supplementary Material

Supplementary Information
