# Supplementary material for: Sec16A is critical for both conventional and unconventional secretion of CFTR
Source: Sci Rep. 2017 Jan 9;7:39887. doi: 10.1038/srep39887 (PMC5220342; doi:10.1038/srep39887)
Supplement: Supplementary Information [file srep39887-s1.pdf]

Supplementary Information

Sec16A is critical for both conventional and unconventional secretion of CFTR

He Piao[1], Jiyoon Kim[1], Shin Hye Noh[1], Hee-Seok Kweon[2], Joo Young Kim[1], and Min Goo Lee[1]

1. Department of Pharmacology, Brain Korea 21 PLUS Project for Medical Sciences, Severance Biomedical Science Institute, Yonsei University College of Medicine, Seoul 03722, Korea.

2. Nano-Bio Electron Microscopy Research Group, Korea Basic Science Institute, Cheongju 28119, Korea

Correspondence: Min Goo Lee, Department of Pharmacology, Yonsei College of Medicine, 50-1 Yonsei-ro, Seodaemun-Gu, Seoul 03722, Korea, Tel: +82 2 2228 1737; Fax: 82 2 313 1894; E-mail: mlee@yuhs.ac

Key Words: Sec16A, unconventional secretion, CFTR, GRASP, IRE1α

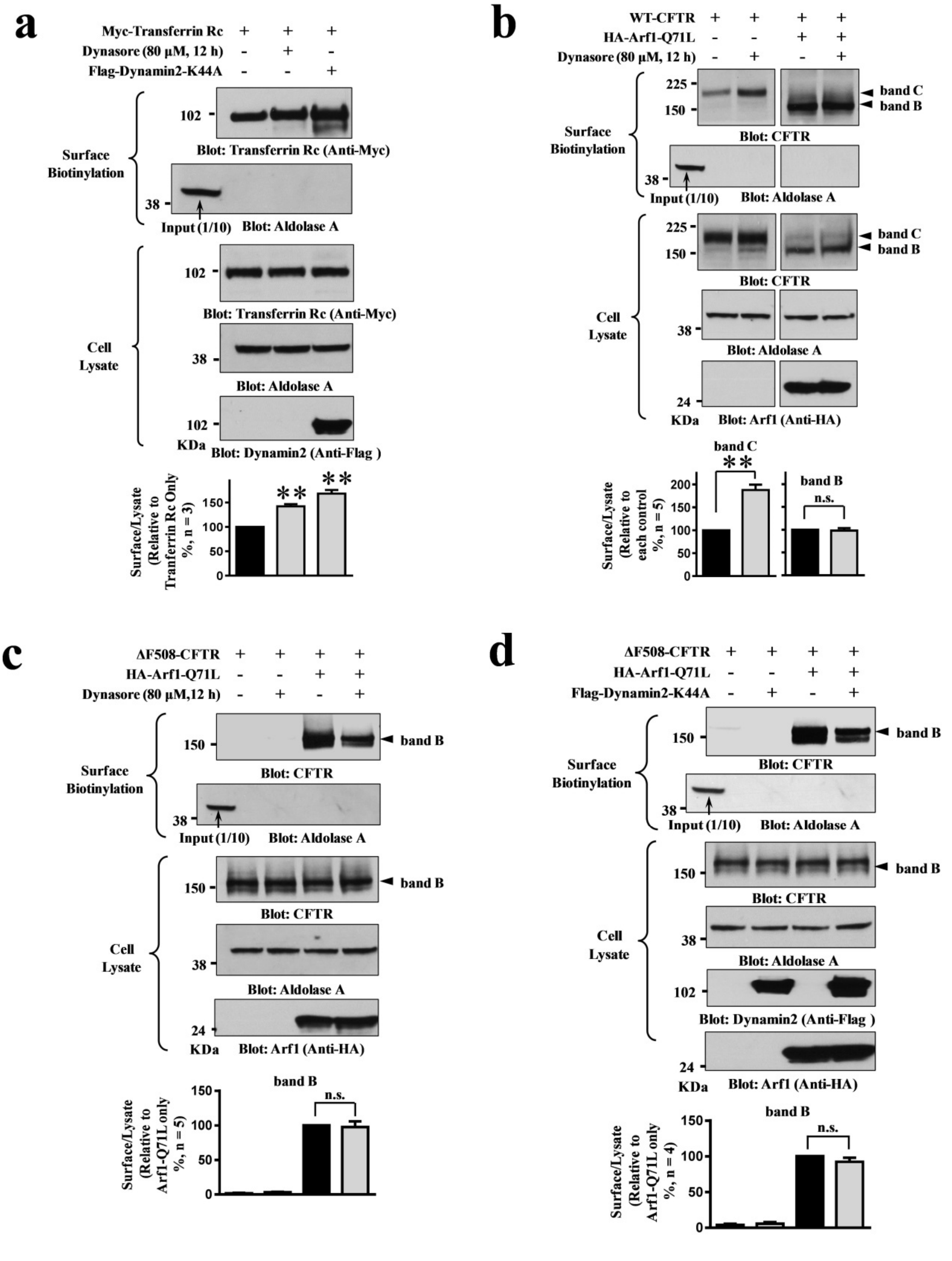

Figure S1. Inhibition of internalization does not affect Arf1-Q71L-mediated surface expression of core-glycosylated CFTR. HEK293 cells were transfected with plasmids expressing transferrin receptor or CFTR, and a surface biotinylation assay was performed 24 h after transfection. Some cells were treated with dynasore or cotransfected with the dynamin2-K44A plasmid to inhibit the dynamin-mediated internalization of surface proteins. (**a**) Dynasore and dynamin2-K44A increased the cell-surface expression of transferrin receptor (Rc) (**b**) Dynasore increased surface expression of band C form of wild-type CFTR; however, did not affect the Arf1-Q71L-mediated surface expression of band B form of wild-type CFTR. (**c, d**) Surface biotinylation of ΔF508-CFTR. Dynasore (**c**) and dynamin2-K44A (**d**) neither induced surface expression of ΔF508-CFTR, nor affected the Arf1-Q71L-mediated surface expression of ΔF508-CFTR. The results of multiple experiments are summarized under each blot. $^{**}P < 0.01$, n.s.: not significant.

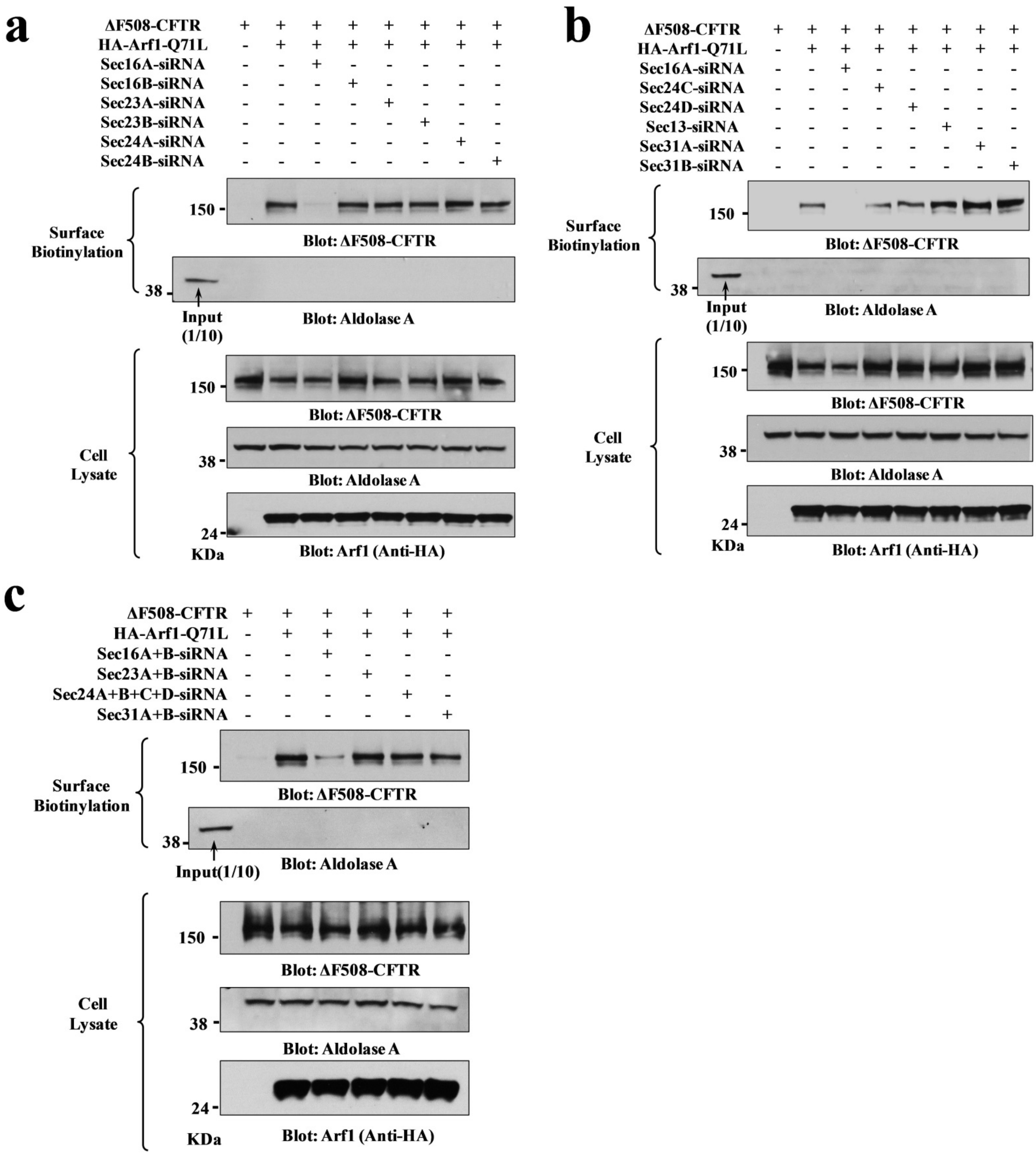

Figure S2. Effects of COPII depletion on the Arf1-Q71L-induced cell-surface expression of ΔF508-CFTR.
(**a, b**) Representative surface biotinylation assays of single gene knockdown. Results of multiple experiments are summarized in Fig. 1e. (**c**) Representative surface biotinylation assays of the combinatorial gene knockdown on the same gene family. Results of multiple experiments are summarized in Fig. 1f. Knockdown of Sec23, Sec24, Sec13, and Sec31 did not affect the unconventional cell-surface transport of ΔF508-CFTR.

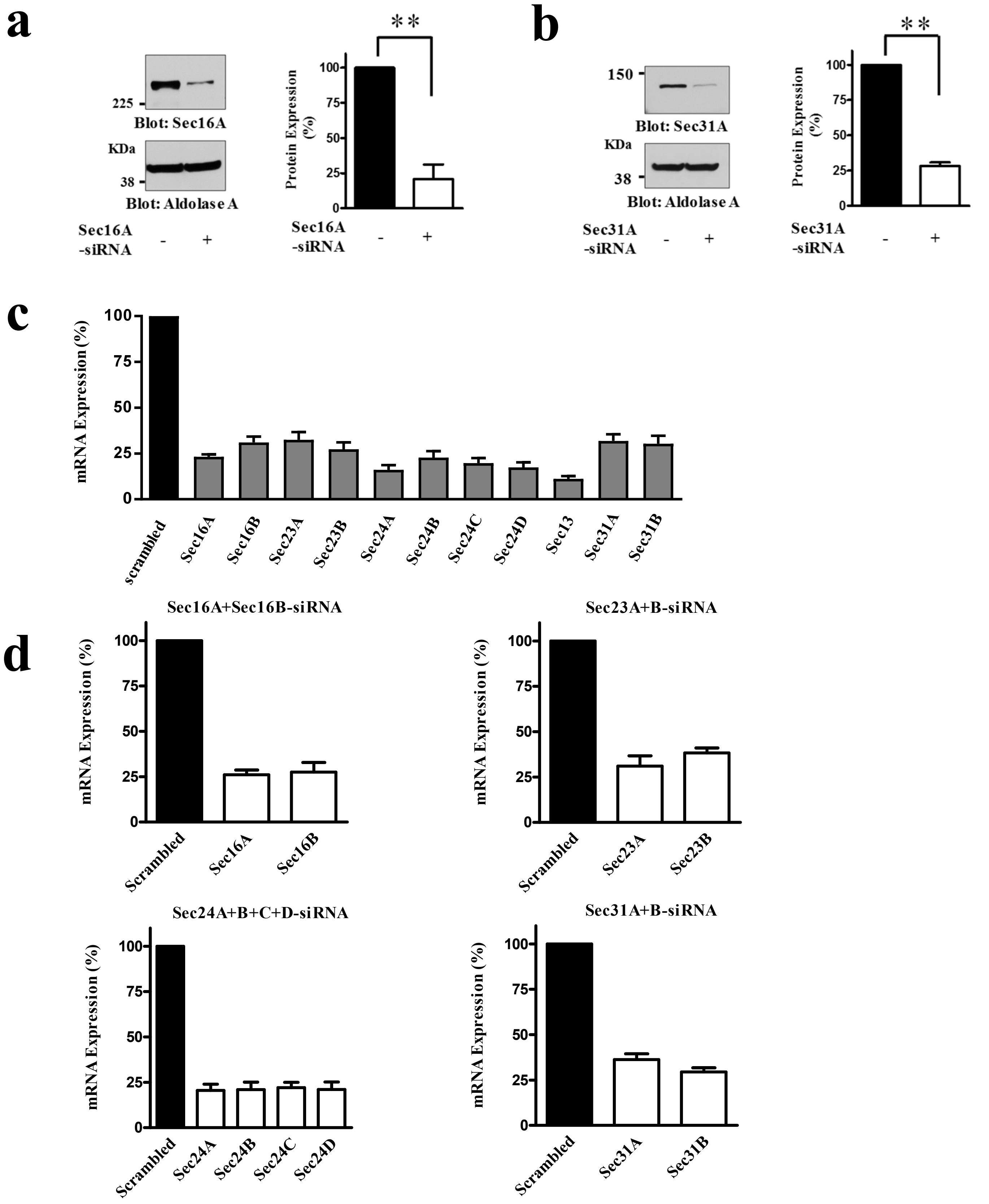

Figure S3. Depletion of Sec16A and core COPII components by siRNA.
(**a, b**) Protein samples were prepared from HEK293 cells 48 h after transfection with each siRNA. A 70–90% depletion of Sec16A (**a**, mean ± SEM, n = 3) and Sec31A (**b**, mean ± SEM, n = 3) was confirmed by immunoblotting, (**c**) mRNA quantitation of all human isoforms of Sec16 and core COPII components including Sec23, Sec24, Sec13, and Sec31 under conditions of single gene knockdown. (**d**) mRNA quantitation of all human isoforms of Sec16 and core COPII components under conditions of the combinatorial gene knockdown on the same gene family (mean ± SEM, n = 3). **$P < 0.01$. mRNA samples were prepared from HEK293 cells 48 h after transfection with each siRNA.

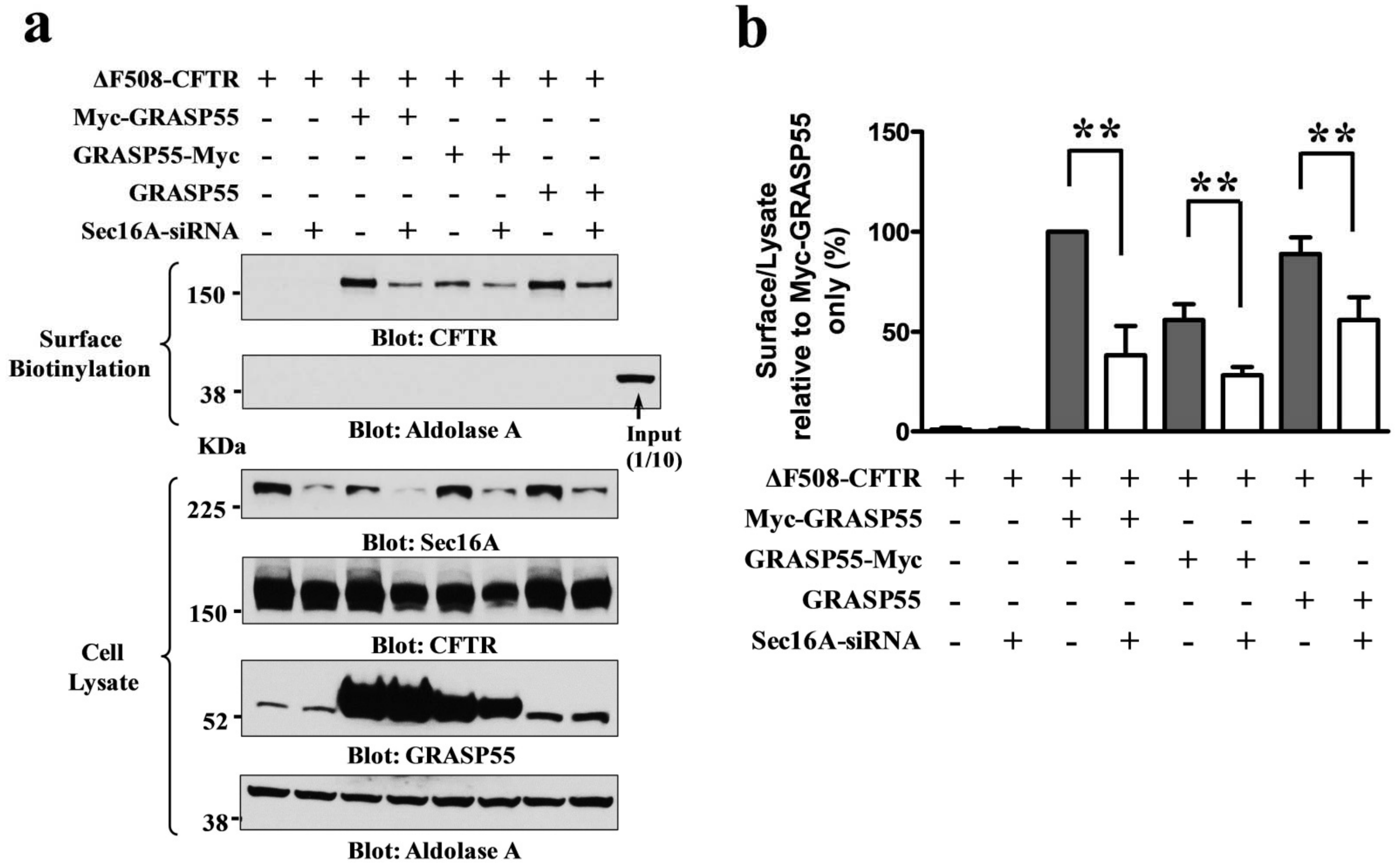

Figure S4. Sec16A is required for GRASP-mediated unconventional secretion of ΔF508-CFTR.
(**a**) Surface biotinylation assays were performed in HEK293 cells after the induction of unconventional secretion of ΔF508-CFTR by GRASP55 overexpression. The cells were pretreated with scrambled or Sec16A-specific siRNAs (100 nM) 24 h before plasmid transfection. A representative surface biotinylation assay is shown. (**b**) Quantification of multiple experiments (mean ± SEM, n = 3) are summarized. Three different GRASP55 variants (Myc-GRASP55, Myc-tagging at the N-terminus of GRASP55; GRASP55-Myc, Myc-tagging at the C-terminus of GRASP55; and GRASP55 without any tag) induced the cell-surface expression of ΔF508-CFTR, which was significantly reduced by Sec16A silencing. *$P < 0.05$, **$P < 0.01$: difference from control.

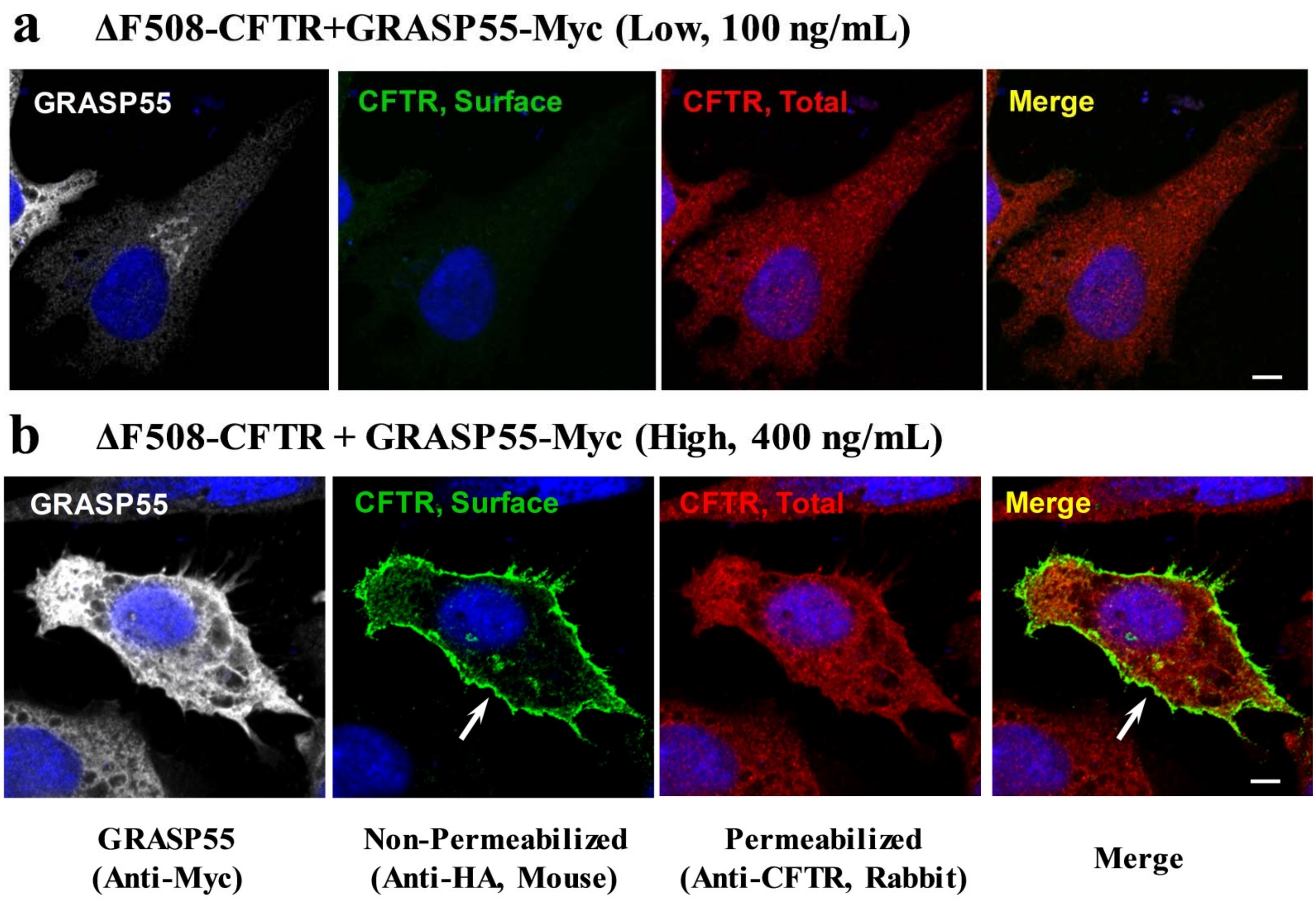

Figure S5. Cell-surface expression of ΔF508-CFTR induced by a high level of GRASP55 expression.
(**a, b**) Extracellular loop HA-tagged ΔF508-CFTR was expressed in HeLa cells with a low level (**a**, 100 ng/mL plasmid transfection) or a high level (**b**, 400 ng/mL plasmid transfection) of GRASP55-Myc coexpression. CFTR at the cell surface was immunostained with anti-HA antibodies before membrane permeabilization (green), and then the total CFTR was stained with anti-R4 CFTR antibodies after permeabilization (red). Arrowheads indicate surface expression of ΔF508-CFTR. A high level of GRASP55 coexpression induced the cell-surface expression of ΔF508-CFTR. Three independent experiments showed similar results. Scale bar: 5 μm.

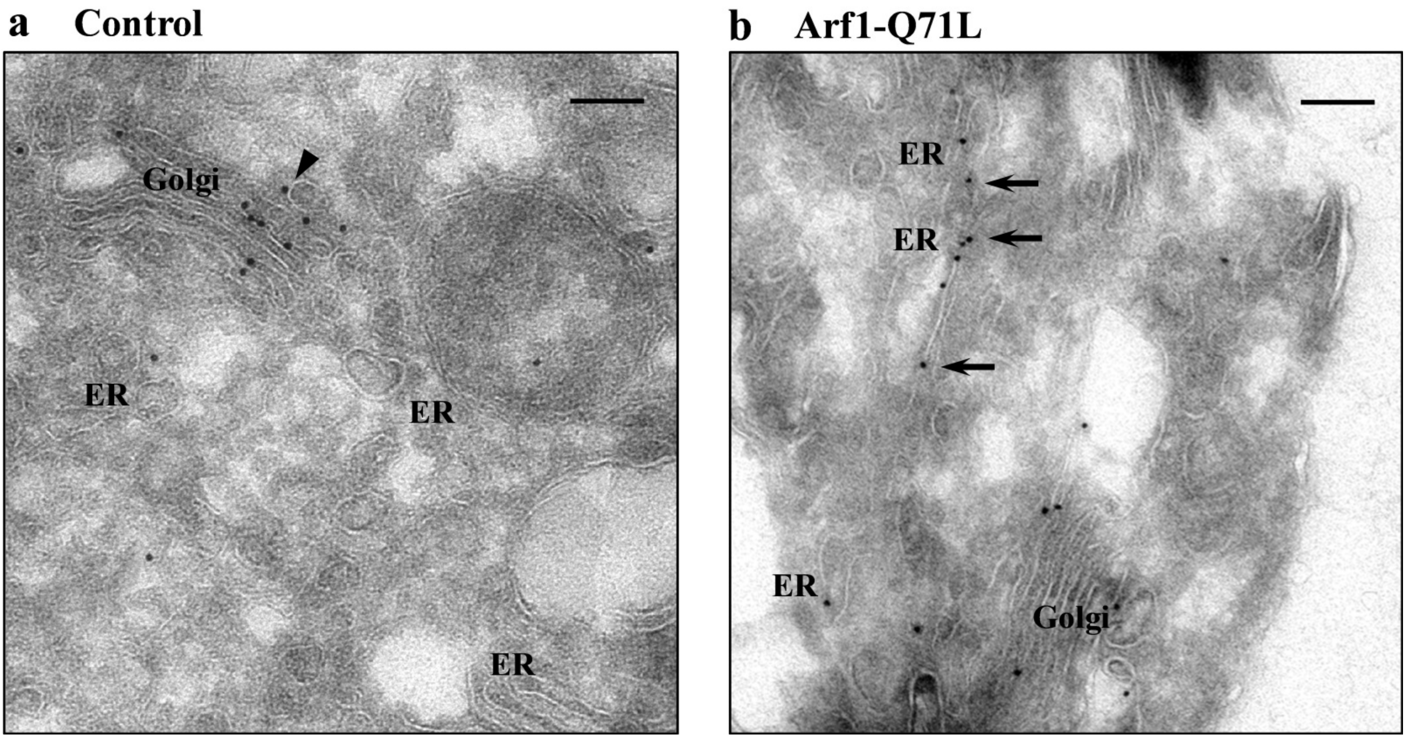

Figure S6. Immunoelectron microscopy of GRASP55.
(**a**) Transmission electron microscopy was performed in HeLa cells after immune-gold labeling of GRASP55 as described in the Materials and Methods. GRASP55 was principally localized in the Golgi (arrowhead).
(**b**) Representative electron microscopy image showed the Arf1-Q71L induced the redistribution of GRASP55 into the ER (arrows). Four independent experiments showed similar results. Scale bar: 200 nm.

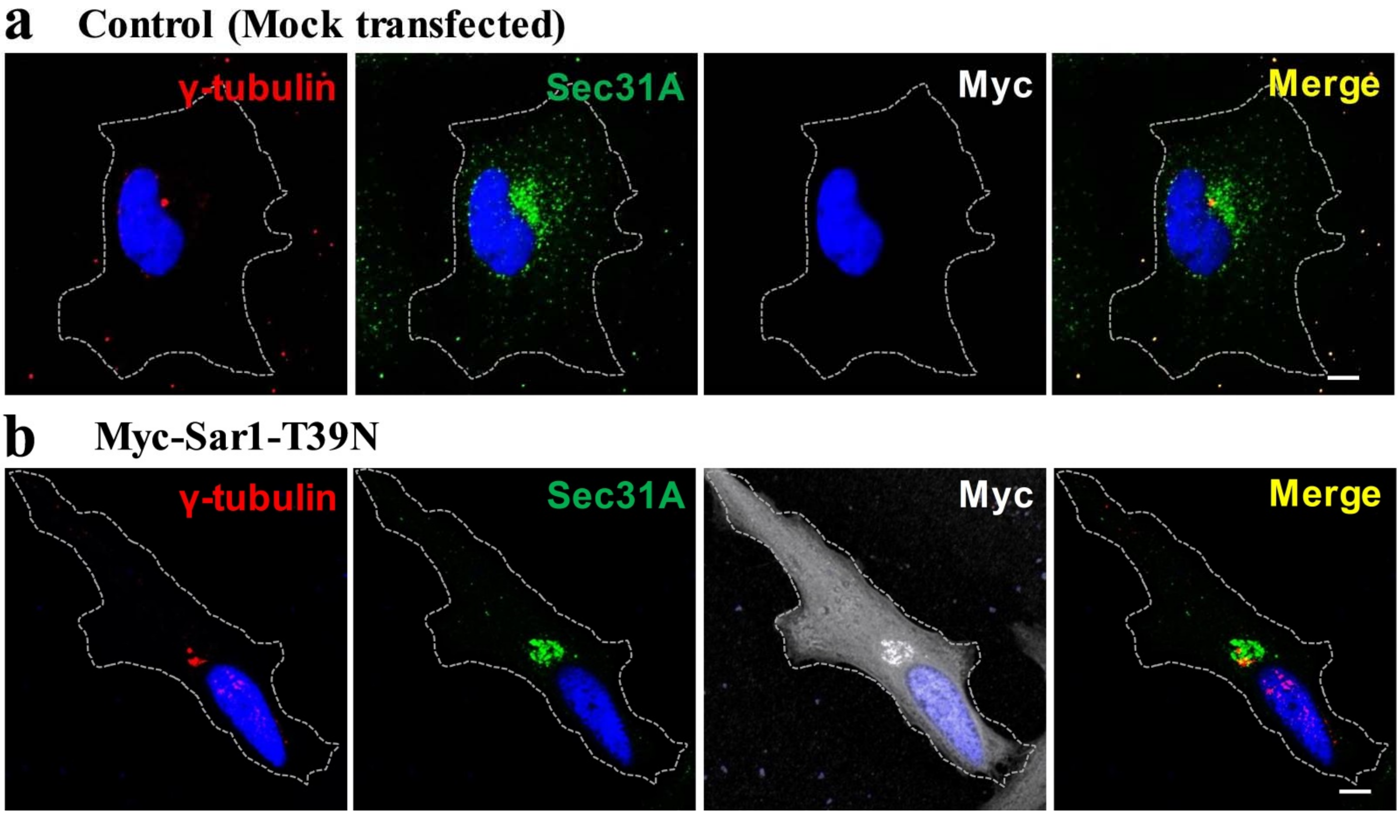

Figure S7. The cellular localization of Sec31A during ER-to-Golgi blockade.
The cellular localization of Sec31A was compared with that of γ-tubulin, a marker of the microtubule-organizing center (MTOC) in HeLa cells. Sec31A and γ-tubulin are co-stained in control cells (**a**) and in cells which were cotransfected with Sar1-T39N to induce ER-to-Golgi blockade (**b**). Sec31A was concentrated near the MTOC in cells with ER-to-Golgi blockade. Scale bar: 5 μm.

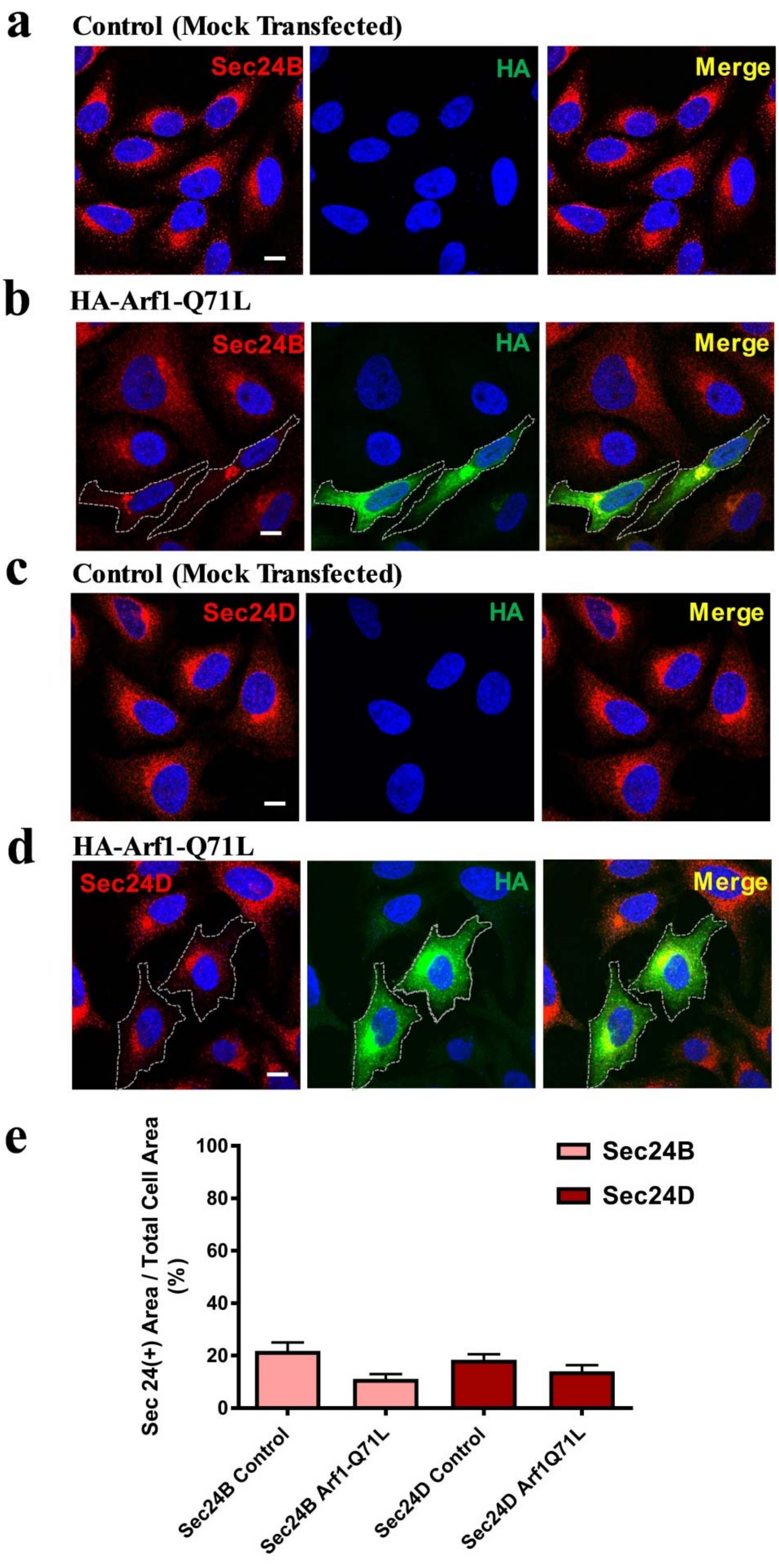

Figure S8. ER-to-Golgi blockade does not relocalize Sec24B or Sec24D.
(**a-d**) The cellular localization of core COPII components Sec24B and Sec24D was confirmed in control cells and in cells treated with Arf1-Q71L. (**e**) Quantification of the ratio of the Sec24B (+) or Sec24D (+) area versus the total cell area in multiple experiments (mean ± SEM, n ≥ 5, each comprising analyses of 5–10 cells) are summarized. Arf1-Q71L induced ER-to-Golgi blockade caused no significant alterations of COPII components' localization. Scale bar: 5 µm.
